# Integrative analysis of single-cell and microarray data reveals SPI1-centered macrophage regulatory signatures in ulcerative colitis

**DOI:** 10.3389/fgene.2025.1617834

**Published:** 2025-09-05

**Authors:** Yeqing Yu, Chengwei Yu, Shanshan Dai, Yixu Liu, Lanlan Hu, Weimin Lu

**Affiliations:** Affiliated Hospital of Nanjing University of Chinese Medicine, Nanjing, China

**Keywords:** single-cell RNA (scRNA) sequencing, SPI1, macrophage, ulcerative colitis, gene regulatory network (GRN)

## Abstract

**Background:**

Ulcerative colitis (UC) is a type of inflammatory bowel disease (IBD) marked by persistent inflammation and ulceration of the colonic mucosal lining. Macrophages regulate intestinal inflammation through distinct polarization profiles. Emerging evidence indicates that the transcription factor SPI1 is a critical regulator of macrophage activity and contributes to both the initiation and progression of UC.

**Methods:**

In this study, single-cell RNA sequencing (scRNA-seq) was conducted to profile the transcriptomic landscape of macrophages in the intestinal tissues of UC patients. A gene regulatory network (GRN) was constructed using pySCENIC, which identified SPI1 as a distinct transcriptional regulator involved in macrophage activation. To pinpoint key downstream targets of SPI1, microarray data were analyzed through a combination of weighted gene co-expression network analysis (WGCNA), differential expression (DE) analysis, and several machine learning algorithms, including LASSO, Recursive feature elimination with a random forest classifier (RFE-RF), and Support Vector Machine-Recursive Feature Elimination (SVM-RFE). An *in vitro* model of M1-polarized macrophages was then established, and Western blot (WB) was used to assess the protein expression of SPI1. SPI1 was then silenced using siRNA, and its impact on macrophage polarization was evaluated using flow cytometry and ELISA.

**Results:**

GRN analysis results suggest that the SPI1(+) regulon regulates macrophage activation in UC. Using WGCNA on microarray data, we identified key downstream regulatory target genes, specifically IRAK3, IL1RN, CD55 and PEA15, based on microarray data. Their potential as biomarkers was subsequently validated through several machine learning algorithms. *In vitro* experiments demonstrated elevated expression of SPI1 in M1-polarized macrophages, as confirmed by WB analysis. Flow cytometry and ELISA analyses revealed that SPI1 silencing inhibited M1 macrophage polarization.

**Conclusion:**

This study identified SPI1 as a potential key transcription factor involved in macrophage polarization in UC, possibly exerting its regulatory effects through IRAK3, IL1RN, CD55 and PEA15. These findings offer a novel perspective on the molecular mechanisms underlying intestinal inflammation in UC.

## Graphical Abstract

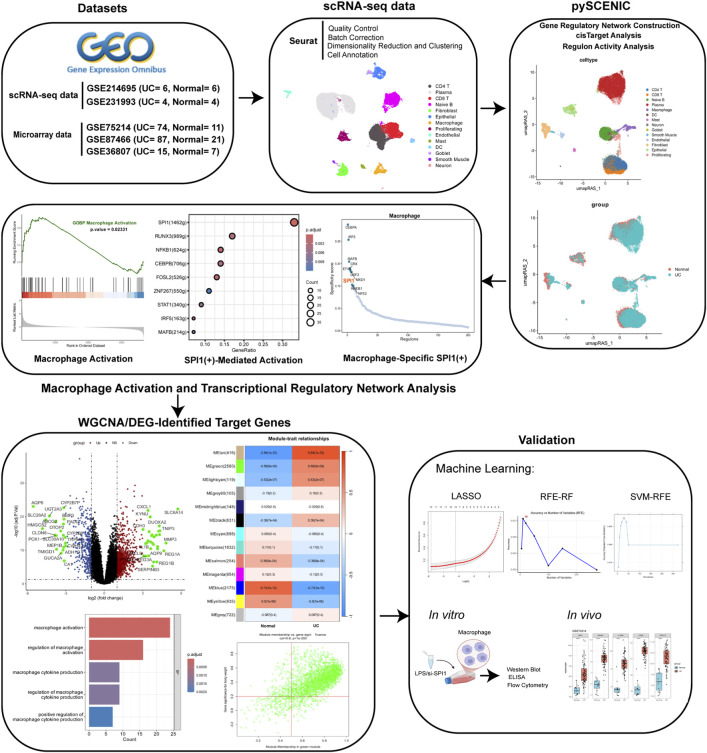

## 1 Introduction

Ulcerative colitis (UC) is a chronic inflammatory bowel disease (IBD) marked by episodes of abdominal pain, diarrhea, and rectal bleeding. Typical endoscopic features include mucosal erythema, edema, and ulceration (Gore et al., 1984; van der Post et al., 2019). In recent years, its incidence has risen steadily, posing UC a growing concern for gastrointestinal health. As the disease advances, inflammation may progress to cryptitis and crypt abscesses, and in severe cases, can result in intestinal strictures or perforation. Persistent and recurrent inflammation over time may drive epithelial-to-mesenchymal transition (EMT), significantly elevating the risk of colitis-associated cancer (CAC) (Bopanna et al., 2017).

As a crucial element of the innate immune system, macrophages are fundamentally involved in the pathogenesis of UC. Research indicates that the extent of intestinal macrophage infiltration is positively associated with UC severity (Mowat and Bain, 2011), and excessive infiltration can compromise the integrity of the intestinal mucosal barrier (Liang et al., 2022). Activated macrophages modulate the inflammatory milieu of the gut. Specifically, overpolarization of pro-inflammatory M1 macrophages or suppression of anti-inflammatory M2 macrophages markedly increases the production of IL-6, leading to the initiation and exacerbation of UC (Zhou et al., 2019; Xu et al., 2022). Furthermore, the accumulation of M1 macrophages in the intestinal lamina propria leads to TNF-α–mediated disruption of epithelial tight junctions, fostering ongoing and recurrent inflammation (Lissner et al., 2015).

SPI1 functions as an important regulator of myeloid cell activity, especially in the context of macrophage activation. Evidence indicates that SPI1 facilitates the differentiation of peripheral blood monocytes into macrophages and modulates their functional properties by influencing M1/M2 polarization (Jego et al., 2014). Moreover, SPI1 interacts synergistically with HOXA3 to dynamically regulate this polarization, thereby contributing to tissue repair and wound healing processes (Al Sadoun et al., 2016). These findings suggest that SPI1 may be involved in the pathogenesis of UC through its regulation of intestinal macrophage polarization, ultimately shaping the course of intestinal inflammation.

The development of single-cell RNA sequencing (scRNA-seq) has opened new avenues for exploring cellular diversity, allowing for detailed investigation of immune cells in multifaceted biological contexts. Using the pySCENIC pipeline, we performed gene regulatory network (GRN) analysis by integrating co-expression patterns from scRNA-seq data with regulator inference, leading to the identification of key differential transcription factors. This approach accurately characterized transcriptional regulatory units specific to macrophage subsets from a network topology perspective (Van de Sande et al., 2020). By combining Weighted Gene Co-expression Network Analysis (WGCNA) with differential expression (DE) analysis, we improved the accuracy of identifying core target genes regulated by transcription factors.

To investigate the regulatory networks involving macrophage transcription factors and their downstream genes in UC, we analyzed two scRNA-seq datasets along with three microarray datasets. The insights gained contribute to a better comprehension of UC’s molecular basis and may aid in the advancement of tailored treatment strategies.

## 2 Methods

### 2.1 Data collection

All datasets were retrieved from the Gene Expression Omnibus (GEO) database (http://www.ncbi.nlm.nih.gov/geo). ScRNA-seq data were obtained from two datasets: GSE214695 (Garrido-Trigo et al., 2023), which includes samples from 6 healthy individuals and 6 patients with active UC, and GSE231993 (Du et al., 2023), containing 4 healthy controls and 4 active UC cases. Microarray datasets were derived from three datasets: GSE87466 (21 healthy controls and 87 active UC samples) (Li et al., 2018), GSE75214 (11 healthy controls and 74 active UC samples) (Vancamelbeke et al., 2017), and GSE36807 (7 healthy controls and 15 active UC samples) (Montero-Meléndez et al., 2013). In patients with active UC, tissue samples were taken from inflamed regions of the colon, while in the control group, samples were obtained from the normal colonic tissue of healthy individuals. Table 1 shows the accession numbers, platforms, and other details of the datasets.

**TABLE 1:** Basic information of datasets involved in the study.

Datasets	Normal	Active UC	Location	Applications	References (PMID)
GSE214695	6	6	Colon	ScRNA-seq analysis	37495570
GSE231993	4	4	Colon	ScRNA-seq analysis	37344477
GSE87466	21	87	Colon	WGCNA & DE analysis	29401083
GSE75214	11	74	Colon	WGCNA & DE analysis	28885228
GSE36807	7	15	Colon	Validation	24155895

### 2.2 Materials and reagents

RAW264.7 cells were obtained from the Cell Bank of the Chinese Academy of Sciences (Shanghai, China). They were maintained in DMEM supplemented with 10% fetal bovine serum and 1% penicillin-streptomycin. Cell cultures were incubated at 37 °C in a humidified atmosphere containing 5% CO_2_. ELISA kit for IL-1β (ZC-37974, Zhuocai, China); ELISA kit for IL-10 (ZC-37962, Zhuocai, China); SPI1 antibody (55100-1-AP, Proteintech, China); iNOS antibody (22226-1-AP, Proteintech, China); Arg1 antibody (16001-1-AP, Proteintech, China); Tubulin antibody (80762-1-RR, Proteintech, China); Anti-rabbit IgG (H + L) (14780, Cell Signaling Technology, United States).

### 2.3 Analysis of scRNA-seq data

We conducted single-cell transcriptome analysis on the GSE214695 and GSE231993 datasets, utilizing the Seurat R package (version 5.1.0) for data processing. ScRNA-seq data underwent quality control filtering to retain cells with 300–6,000 detected genes, <10% mitochondrial reads, >1,000 UMIs, and total counts below the 97th percentile (Supplementary Figure S1). Data processing followed standard Seurat workflow including normalization, variable gene identification, and data scaling (Supplementary Figure S2). After initial dimensionality reduction via PCA, the Harmony algorithm was applied to mitigate batch effects (Supplementary Figures S3, S4). An SNN graph was constructed based on the top 40 harmony dimensions, and Louvain clustering at 0.4 resolution yielded 18 distinct cell clusters (Supplementary Figure S5). Finally, UMAP was used for two-dimensional visualization of cellular heterogeneity. Afterwards, we performed differential expression analysis on our Harmony-integrated scRNA-seq data using the FindAllMarkers function in Seurat, applying Wilcoxon rank sum test with stringent filtering criteria (logfc.threshold = 0.25, min.pct = 0.25, only.pos = TRUE). This approach enabled us to identify cluster-specific marker genes that facilitated accurate cell type annotation and provided valuable insights for subsequent functional characterization of cell subpopulations.

### 2.4 Identifying differential regulons with pySCENIC

We leveraged pySCENIC methodology to elucidate GRN at single-cell resolution (Supplementary Figure S6). The analytical pipeline commenced with GRNboost2 algorithm implementation to swiftly detect potential regulatory interactions by examining transcription factor and target gene co-expression patterns within single-cell transcriptomic datasets (Van de Sande et al., 2020). We then incorporated the human promoter motif repository and used RcisTarget to identify binding sites within promoter regions, filtering for sequence-validated interactions to establish robust regulons. Regulatory influence across cellular populations was quantified via area under the curve cell-level (AUCell)-derived activity scores, complemented by PCA dimensionality reduction to offer alternative visualization of regulatory landscapes. The resultant cell-type-specific transcriptional networks illuminated distinctive molecular governance across diverse cellular states and developmental trajectories, providing crucial insights into heterogeneity and disease mechanisms. Moreover, we deployed cell-specific index (CSI) to characterize regulatory modules, with transcription factor relationships assessed through Pearson correlation and visualized.

To identify cell type-specific regulons, we first computed a binary indicator matrix representing the presence of each cell type across all cells. Using the regulon activity score matrix (rasMat), we then calculated the Regulon Specificity Score (RSS) for each regulon across different cell types. Specifically, RSS was defined as 1− Jensen-Shanno Divergence (JSD). 1− JSD between the regulon’s activity distribution and the idealized distribution of each cell type. A higher RSS indicates stronger specificity of a regulon to a particular cell type. The top-ranked regulons for each cell type were visualized based on their RSS values.

### 2.5 Pseudobulk-based differential expression and SPI1 regulon activity analysis in macrophages

To investigate transcriptional regulation in UC, macrophage subsets were extracted from both UC and healthy control groups using preprocessed Seurat objects. Due to the sparsity of scRNA-seq data, a pseudobulk strategy was applied by aggregating gene expression profiles at the sample level, resulting in a gene-by-sample count matrix. Differential gene expression analysis was conducted using the DESeq2 package following median ratio normalization. Genes with at least 10 normalized counts in three or more samples per group were retained. Differentially expressed genes between UC and normal groups were identified based on a negative binomial generalized linear model. Gene set enrichment analysis (GSEA) was performed using the clusterProfiler R package (version 4.12.6) (Wu et al., 2021). Enriched pathways were then mapped onto the previously constructed transcription factor regulons.

To assess SPI1 activity in macrophages, we extracted its regulon target genes and calculated their average expression per cell using Seurat’s AddModuleScore (). This average expression served as an activity score reflecting SPI1 regulatory strength, which we visualized on UMAP and compared across macrophage subsets. Specifically, we assessed the expression activity of each SPI1 regulon target gene within macrophages and aggregated these measurements to estimate SPI1’s downstream regulatory impact.

### 2.6 WGCNA-based identification and validation of UC-Associated modules

To investigate the association between gene expression and clinical traits, we employed WGCNA to construct gene co-expression networks (Supplementary Figure S7). Initially, the WGCNA R package (version 1.72.5) was used to calculate pairwise gene correlations using Pearson’s correlation coefficient, resulting in a gene co-expression matrix (Langfelder and Horvath, 2008). To ensure the inclusion of biologically informative genes, we implemented the PVAC filtering strategy, which integrates variance, median absolute deviation (MAD), coefficient of variation (CV), and expression levels. Genes were retained for downstream analysis only if they exhibited high expression, fell within the top 75% in terms of variability, and had a minimum expression value greater than 1. Next, the pickSoftThreshold function was applied to determine the optimal soft threshold (β) for constructing a signed network, adhering to the scale-free topology criterion. This allowed us to construct a signed, weighted gene co-expression network that emphasizes positive correlations and conforms to a scale-free architecture, an important feature for identifying biologically meaningful modules associated with immune activation in UC. Modules were identified through hierarchical clustering and dynamic tree cutting, followed by module merging. Following module detection, we assessed the association between each module and UC by calculating the Pearson correlation between module eigengenes and the disease phenotype.

To identify WGCNA modules associated with SPI1 regulation, SPI1 target genes were first intersected with the set of expressed genes. The number of SPI1 targets within each module was then quantified. Subsequently, a hypergeometric test was conducted to evaluate the significance of target gene enrichment in each module. Modules exhibiting significant enrichment were deemed closely linked to SPI1 and were selected for downstream analyses. The module membership (MM) of each gene was evaluated, which is defined as the correlation between the gene and its module eigengene. The gene significance (GS) of a gene was also calculated, which is defined as the correlation with the UC phenotype. Genes with MM > 0.5 and GS > 0.2 were visualized via scatter plots, and those meeting both criteria were identified as candidate hub genes. Candidate hub genes identified from the module in one dataset were used to reconstruct a module eigengene in the other dataset via PCA. The association between the reconstructed eigengene and disease status was evaluated using Welch’s t-test. A significant difference in eigengene values supported the robustness of the association between the core module genes and UC.

To evaluate the reproducibility of co-expression modules, we conducted a module preservation analysis using WGCNA. Modules detected in GSE87466 were assessed for preservation in GSE75214 with 200 permutations and a signed network approach. Preservation strength was measured by the Zsummary statistic, where values above 10 indicate strong preservation, between 2 and 10 moderate preservation, and below 2 weak or no preservation. This analysis confirmed that key modules were consistently preserved across independent datasets, demonstrating their robustness.

### 2.7 Acquisition of DE analysis and enrichment analysis

Two microarray datasets, GSE87466 and GSE75214, were analyzed to identify differential expressed genes. A series of quality control procedures were applied to both datasets, including normalization using the normalizeBetweenArrays function, gene annotation and integration, and the detection of potential outliers through PCA (Supplementary Figures S8–S11). Following preprocessing, differentially expressed genes were identified using the limma R package (version 3.60.4), with the filtering criteria set to |logFC| ≥ 1 and a p-value <0.05 (Ritchie et al., 2015). Volcano plots were generated using the ggplot2 R package (version 3.5.1) to visualize the differential expressed genes. To investigate their biological relevance, GO enrichment analysis was conducted using the clusterProfiler package, focusing on processes associated with macrophage activation.

### 2.8 Machine learning screening

To identify potential downstream target genes regulated by transcription factors, LASSO, Recursive feature elimination with a random forest classifier (RFE-RF), and Support Vector Machine-Recursive Feature Elimination (SVM-RFE) were systematically applied. The training dataset was created by merging GSE75214 and GSE87466, with batch effects corrected using the sva R package (version 3.52.0) (Supplementary Figure S24). GSE36807 was designated as the independent validation set.

Given the high dimensionality of gene expression data, LASSO regression was first employed to perform variable selection and dimensionality reduction. Gene expression profiles were standardized, and LASSO logistic regression was conducted with 10-fold cross-validation to identify the optimal regularization parameter (λ) using the λ.1se criterion (Friedman et al., 2010). Genes with non-zero coefficients at this penalty level were considered candidate biomarkers with discriminative potential between UC and control samples. To ensure the robustness of selected features, stability selection was applied using the stabsel function, with a selection probability cutoff of 0.7 and PFER control (Hofner et al., 2015). Genes consistently selected across subsamples were considered stable and reliable predictors.

RFE-RF was implemented using the caret R package (version 6.0.94) (Liaw and Wiener, 2002), incorporating repeated 10-fold cross-validation to identify the optimal feature subset. Feature selection was guided by classification accuracy. A random forest model with 500 trees (ntree = 500) was then trained on the selected features, and variable importance was assessed via the Mean Decrease Gini index. To further confirm the robustness and relevance of the selected features, the Boruta algorithm was applied, with tentative attributes resolved using the TentativeRoughFix () function (Kursa et al., 2010).

In parallel, the SVM-RFE algorithm was employed to identify the most informative gene subset for classification, utilizing the caret package with a linear support vector machine classifier (svmLinear) implemented through the e1071 package (version 1.7.14) (Guyon et al., 2002). Prior to feature selection, gene expression data were row-wise standardized (Z-score normalization). The recursive feature elimination was performed with repeated 10-fold cross-validation (5 repeats) to ensure model stability and reduce overfitting. Various feature subset sizes were evaluated, and the optimal feature set was chosen based on the balance between classification accuracy and error rate.

### 2.9 Diagnostic performances analysis

To assess the diagnostic potential of the target genes, receiver operating characteristic (ROC) curves were generated using the pROC R package (version 1.18.5), and the corresponding area under the curve (AUC) values were computed (Robin et al., 2011). The GSE36087 dataset served as an external validation cohort. The expression levels of the target genes were analyzed in both UC and normal samples from the validation set, and ROC analysis was conducted to further confirm their discriminative ability.

### 2.10 LPS-induced macrophage activation

RAW264.7 macrophages were subjected to M1-like polarization following two passages. Cells were treated with LPS (1 μg/mL) for 24 h to induce M1 polarization. After stimulation, cells were harvested and processed for subsequent assays. Throughout all experiments, untreated cells consistently served as blank controls to establish baseline values.

### 2.11 siRNA-mediated knockdown of SPI1

A small interfering RNA (siRNA) targeting SPI1 was designed and synthesized by Xuzhou General Biotechnology Co., Ltd. To evaluate the impact of SPI1 knockdown, cells were assigned to three groups: (1) control (untreated), (2) si-NC (transfected with scrambled, non-targeting siRNA as a negative control), and (3) si-SPI1 (transfected with siRNA specifically targeting SPI1).

Prior to transfection, cells were plated at 30%–40% confluence in antibiotic-free growth medium and cultured for approximately 24 h. Transient transfection was carried out using Lipofectamine reagent (Tiangen Biochemical Technology Co., Ltd.) following the manufacturer’s protocol.

### 2.12 Western blot (WB)

All WB experiments were performed with three independent biological replicates (n = 3). Cells were lysed in RIPA buffer, and the resulting lysates were clarified by centrifugation. Protein concentrations were measured using the BCA assay and adjusted to ensure consistency among samples. Equal amounts of protein were loaded onto SDS-PAGE gels for separation and subsequently transferred to PVDF membranes. After blocking with 5% skim milk, membranes were incubated overnight with primary antibodies. Following washes, secondary antibodies were applied and incubated for 1 h. Protein signals were detected using an ECL substrate kit and quantified using ImageJ software.

### 2.13 ELISA analysis

Quantitative determination of inflammatory cytokines IL-1β and IL-10 was performed using ELISA methodology. All measurements were performed in triplicate using independent biological samples. Serial dilutions of reference standards were prepared in accordance with the manufacturer’s protocol to establish calibration curves. Absorbance measurements were obtained spectrophotometrically at 450 nm wavelength for all experimental samples, with blank wells serving as the reference baseline for instrument calibration. This standardized analytical approach enabled precise quantification of the target cytokines in the experimental specimens.

### 2.14 Flow cytometry

Flow cytometry experiments were conducted with at least three independent biological replicates. RAW 264.7 cells in the logarithmic growth phase were seeded into 6-well plates at a density of 1 × 10^6^ cells per well. After enzymatic digestion, cells were collected by centrifugation, resuspended in a fluorescent dye working solution, and incubated on ice for 15 min. Prior to analysis, cells were washed and resuspended in PBS. Flow cytometry was subsequently performed for detection.

### 2.15 Statistical analysis

Statistical analysis was performed using GraphPad 8.0 software. A Student’s t-test was used for two-group comparisons. For comparisons among more than two groups, a one-way ANOVA followed by a Tukey’s *post hoc* test was applied. Results are expressed as mean ± standard deviation (SD). Differences were considered statistically significant at *P* < 0.05 and highly significant at *P* < 0.01.

## 3 Results

### 3.1 Single-cell clustering reveals UC-associated remodeling of cellular composition

Although the two scRNA-seq datasets were derived from partially overlapping regions of the gut, we independently analyzed each dataset and observed a consistent increase in macrophage proportions in UC samples (Supplementary Figure S12). Given this consistent trend across both datasets, we integrated them for downstream analyses. This integration not only provides the increased cell numbers and diversity needed for regulatory network inference using pySCENIC, but also enables more comprehensive characterization of cell-type composition in the UC microenvironment.

**FIGURE 1:**
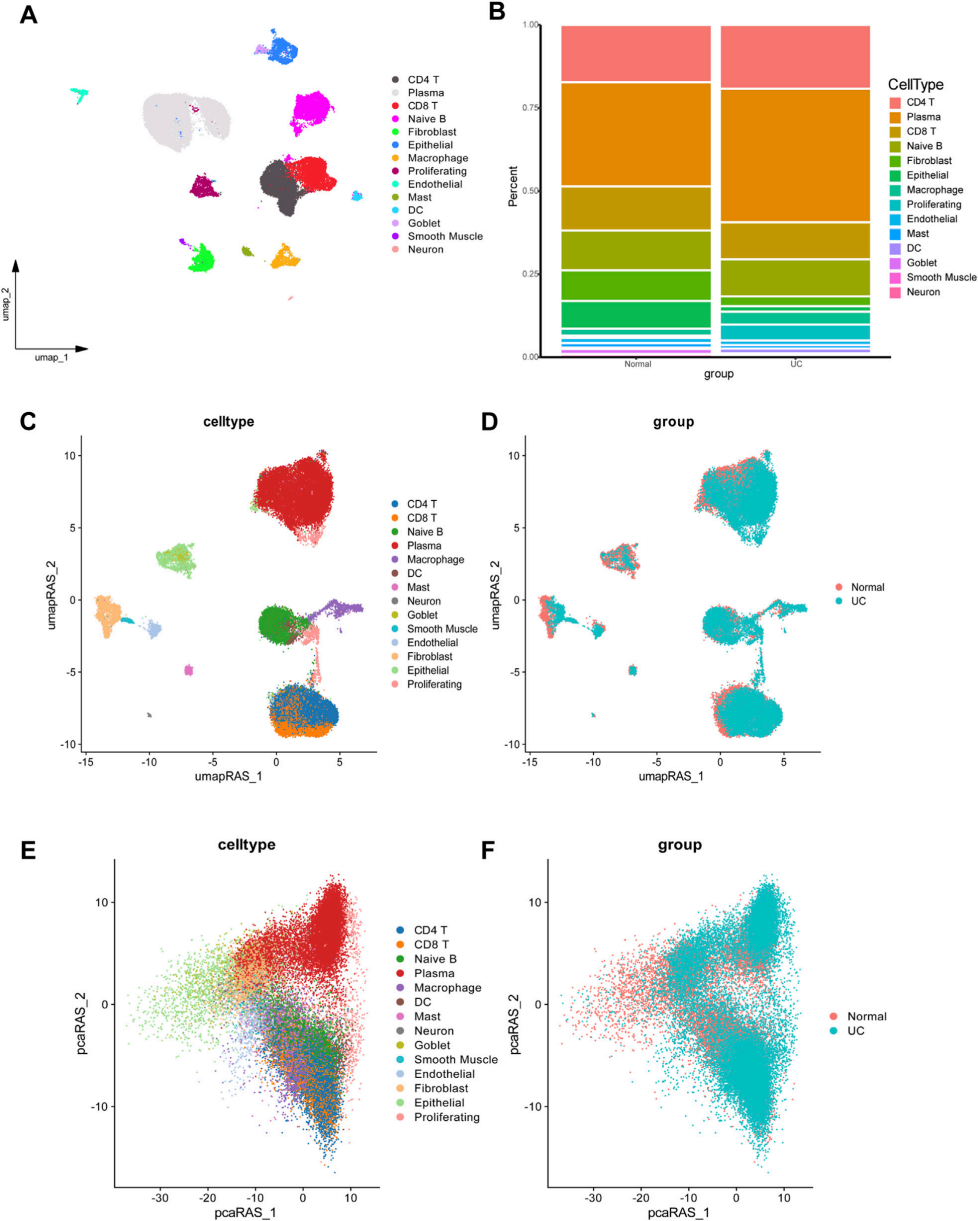
Single-cell RNA-seq analysis data of UC and normal group. **(A)** UMAP plot showing the clustering of different cell types. **(B)** Comparison of cellular composition between UC and normal group. **(C,D)** UMAP dimensionality reduction based on regulon activity scores, providing enhanced visualization of cell type clusters **(C)** and disease-specific differences **(D)**. **(E,F)** Dimensionality reduction plots showing cell type distribution **(E)** and disease status **(F)** based on PCA.

Based on marker gene expression, the integrated scRNA-seq dataset was categorized into 14 distinct cell subpopulations (Figure 1A). Annotation of cell populations was performed using specific markers from the CellMarker database (Supplementary Figure S13). Analysis of cellular composition revealed distinct patterns between groups (Figure 1B). The control group was dominated by plasma cells (31.3%), followed by CD4 T cells (17.2%), CD8 T cells (13.3%), and naive B cells (13.0%), with lower proportions of fibroblasts (9.2%), epithelial cells (8.3%), and macrophages (2.1%). In contrast, UC samples showed significant shifts in cellular distribution. Immune cells expanded, with plasma cells increasing to 40.2%, CD4 T cells to 19.1%, and macrophages to 3.8%. Simultaneously, structural cells declined markedly, with fibroblasts decreasing to 2.9% and epithelial cells to 1.6%. Both CD8 T cells and naive B cells slightly decreased to 11.1% each, while all other cell types collectively comprised less than 11%.

### 3.2 Transcriptional regulatory network analysis in UC

Based on pySCENIC, we identified significantly active regulons. The activity of each regulon within individual cells was evaluated using the AUCell scoring algorithm, followed by UMAP dimensionality reduction clustering based on activity scores across all regulons (Figures 1C,D). Our analysis revealed notable differences in macrophages between UC and normal tissues. Based on the CSI among 401 regulons (TF-target gene pairs) identified by pySCENIC, cells were clustered into 9 modules (Supplementary Figure S14). The average activity of regulons within each module is illustrated in Supplementary Figure S15. Following PCA-based dimensionality reduction, a significant difference in macrophage enrichment between the UC and normal groups was observed (Figures 1E,F), suggesting that macrophages represent key differential cell types distinguishing UC from healthy controls.

### 3.3 Association of SPI1 regulon with UC-related macrophage activation

**FIGURE 2:**
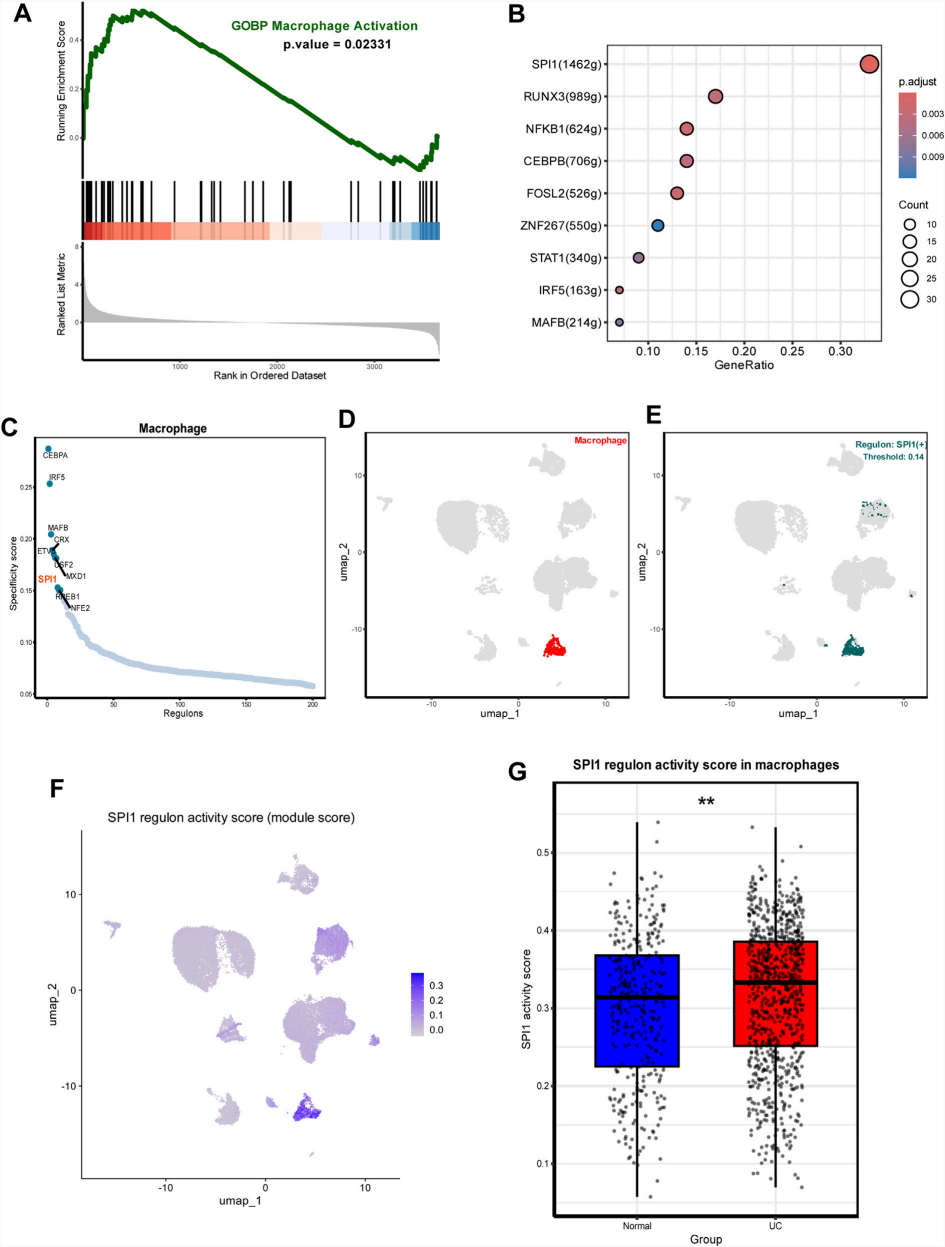
SPI1(+) mainly regulates UC macrophage activation. **(A)** GSEA plots showing significant enrichment of macrophage activation-associated pathway in UC. **(B)** Transcription factors regulating macrophage activation-associated pathway including SPI1 (+). **(C)** Rank for regulons in macrophages based on the RSS. **(D)** Macrophages are highlighted in the UMA; **(E)** Binarized RAS for the top regulon SPI1 on UMAP. **(F)** UMAP showing SPI1 regulon activity across cell types. **(G)** Average expression of SPI1 regulon genes in macrophages. Data are expressed as mean ± SD. *P < 0.05, **P < 0 .01 compared to the other group.

To investigate the molecular mechanisms underlying altered macrophage function in UC, we conducted GSEA to identify signaling pathways enriched in macrophages. The analysis revealed significant enrichment of macrophage activation-associated pathway in UC compared to healthy controls (Figure 2A). To uncover potential upstream regulatory mechanisms driving these changes, we performed transcriptional regulatory network analysis. This revealed that SPI1 and its downstream targets comprised a substantial portion of the identified functional gene sets (Figure 2B). Furthermore, SPI1 regulon activity differed markedly between macrophages from UC patients and those from the control group (Figures 2C–E; Supplementary Table S1). We extracted SPI1 regulon target genes and calculated their average expression per cell using Seurat’s AddModuleScore () function. This average expression score served as a quantitative proxy for SPI1 regulatory strength. Visualization on UMAP embeddings showed heterogenous distribution of SPI1 regulon activity, with macrophages displaying significantly higher scores compared to other cell types (Figure 2F). We also calculated the average expression level of each SPI1 regulon gene within macrophages. Comparison between UC and control groups revealed that SPI1 target genes were significantly upregulated in UC macrophages, further supporting transcriptional activation of SPI1 (Figure 2G). These findings suggest that SPI1 is a key transcription factor involved in regulating macrophage activation in UC.

### 3.4 Genes related to macrophage activation in ulcerative colitis identified by WGCNA and DE analyses

**FIGURE 3:**
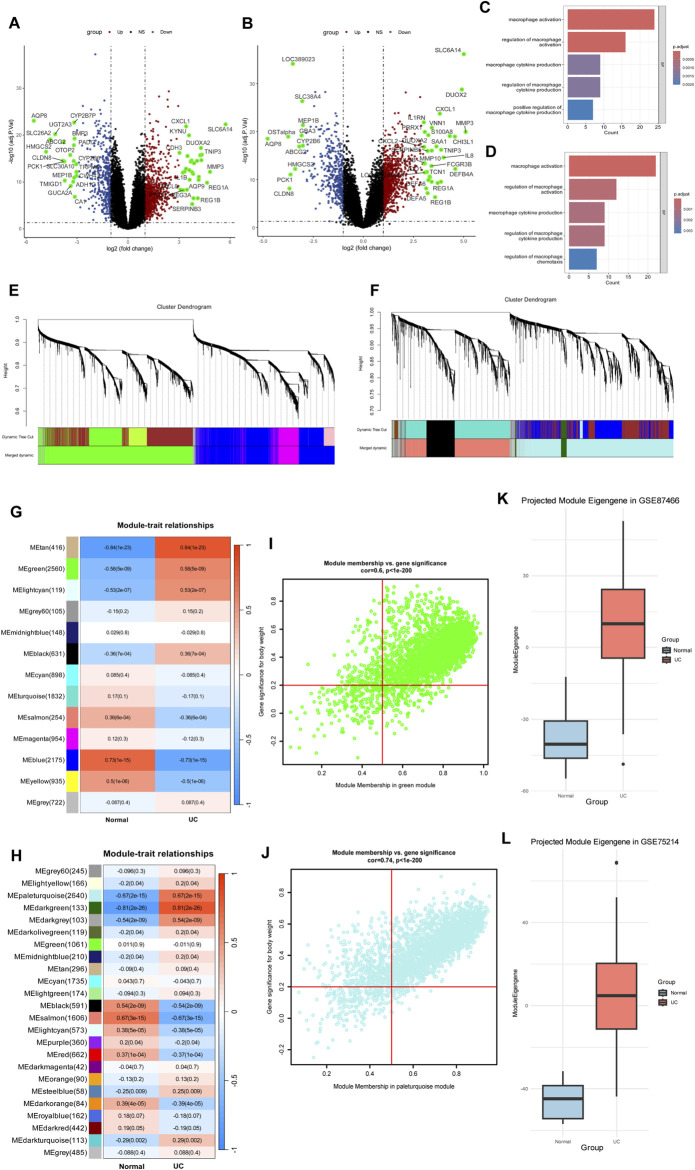
Microarray data identify genes associated with macrophage activation. **(A,B)** Volcano plots in GSE75214 **(A)** and GSE87466 **(B)**. **(C,D)** GO enrichment analysis reveals significant enrichment of macrophage activation pathways. **(E,F)** Cluster dendrograms from WGCNA analysis showing module identification in GSE75214 **(E)** and GSE87466 **(F)**. **(G,H)** Heatmaps showing module-trait relationships between identified gene modules and disease status (Normal vs. UC) in GSE75214 **(G)** and GSE87466 **(H)**. **(I,J)** Module membership versus gene significance correlation plots of green module in GSE75214 **(I)** and paleturquoise module in GSE87466 **(J)**. **(K)** Cross-dataset validation of the green module eigengene from GSE75214 projected into GSE87466. The eigengene showed a strong association with ulcerative colitis (UC) status (Welch’s t-test, t = −13.96, P < 2.2 × 10^−16^). **(L)** Cross-dataset validation of the paleturquoise module eigengene from GSE87466 projected into GSE75214. A significant association with UC was also observed (Welch’s t-test, t = −13.03, P = 1.42 × 10^−14^).

We applied the limma package to analyze two gene expression datasets, GSE87466 and GSE75214 (Figures 3A,B; Supplementary Tables S2, S3). Based on the identified differentially expressed genes, GO enrichment analysis was performed to investigate functional associations (Figures 3C,D). Both datasets revealed a significant enrichment of biological processes associated with macrophage activation.

To further explore the molecular mechanisms underlying UC, we applied WGCNA to the microarray gene expression profiles of both datasets. In GSE87466, one outlier sample (GSM2332144) was excluded based on hierarchical clustering (Supplementary Figure S16). A soft-threshold power of β = 14 was selected to construct a scale-free network (Figure 3F; Supplementary Figure S17). After module merging and refinement, 24 co-expression modules were identified (Figure 3H). Based on the enrichment analysis of SPI1 target genes, we identified the MEpaleturquoise module as significantly enriched and thus selected it for further investigation (Supplementary Figure S18; Supplementary Table S7). This module also exhibited a strong positive correlation with the UC phenotype (cor = 0.67, *P* = 2e−15), suggesting a potential association with macrophage activation in UC (Figure 3H). Genes within this module that met the criteria of MM > 0.5 and GS > 0.2 were defined as core genes (Figure 3J; Supplementary Table S4). In GSE75214, all samples clustered appropriately without any outliers, as shown by hierarchical clustering (Supplementary Figure S19).Similarly, in GSE75214, a soft-threshold of β = 12 was chosen (Figure 3E; Supplementary Figure S20), resulting in 13 modules after dynamic tree cutting and merging (Figure 3G). The MEgreen module was chosen due to its significant enrichment of SPI1 targets and moderate association with UC traits (cor = 0.58, *P* = 5e−9) (Supplementary Figure S21; Supplementary Table S6). Core genes from this module were similarly identified based on MM > 0.5 and GS > 0.2 (Figure 3I; Supplementary Table S5). These gene sets from both datasets were regarded as candidate targets potentially involved in macrophage-mediated pathogenesis of UC.

As shown in Supplementary Figure S22, most modules exhibited Zsummary >2, indicating moderate preservation across the two independent datasets. These findings demonstrate that key co-expression modules are preserved between GSE87466 and GSE75214, indicating cross-dataset consistency in gene co-expression patterns.

To evaluate the robustness of eigengene-trait associations, we performed cross-dataset validation between GSE75214 and GSE87466 through PCA. The association with disease status was tested using Welch’s t-test. The green module eigengene from GSE75214 showed a strong association with UC in GSE87466 (t = −13.96, *P* < 2.2e-16) (Figure 3K). Similarly, the paleturquoise module from GSE87466 was significantly associated with UC when projected into GSE75214 (t = −13.03, *P* = 1.42e-14) (Figure 3L). These results confirm that eigengene–trait relationships are consistent across datasets, supporting the stability of SPI1-enriched gene modules in UC.

### 3.5 Identification and diagnostic assessment of SPI1-Regulated core target genes

**FIGURE 4:**
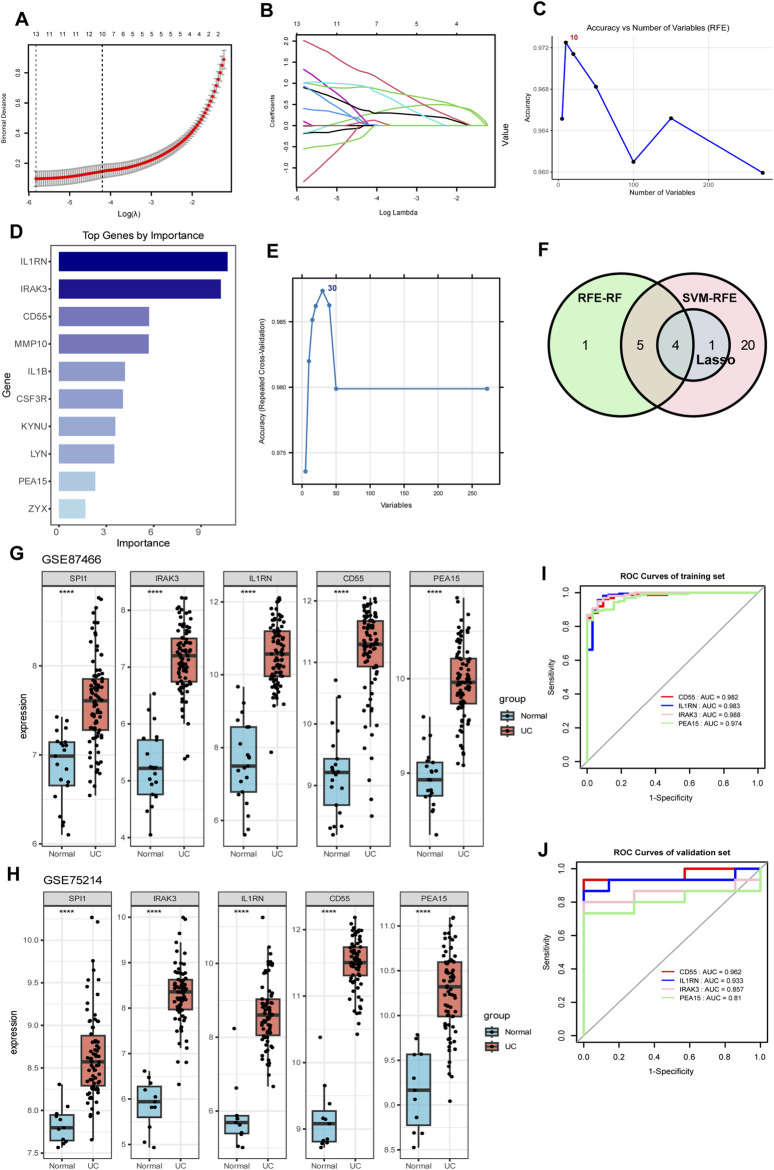
Identification of SPI1-regulated hub genes through the integration of gene set intersection and machine learning approaches. **(A,B)** LASSO regression analysis with cross-validation curve showing the optimal lambda value selection and coefficient profiles for gene selection. **(C)** Accuracy versus number of variables selected using RFE. **(D)** Bar chart of top genes ranked by importance scores. **(E)** SVM-RFE identified a 30-gene signature that achieved the highest classification accuracy. **(F)** Venn diagram illustrating the overlap between genes selected by three machine learning methods (LASSO, RFE-RF, and SVM-RFE). **(G,H)** Expression levels of SPI1, IRAK3, IL1RN, CD55, and PEA15 in two independent datasets GSE87466 **(G)** and GSE75214 **(H)**. **(I)** ROC curves for IRAK3, IL1RN, CD55 and PEA15 in the training dataset. **(J)** ROC curves for IRAK3, IL1RN, CD55 and PEA15 in the validation dataset. ****P < 0.0001 compared to the control group.

We investigated the target genes regulated by SPI1 in macrophages and conducted an intersection analysis with two sets of core genes identified through WGCNA. This analysis revealed 272 overlapping core genes (Supplementary Table S8). To further refine the selection, LASSO regression identified 11 candidate genes with non-zero coefficients at the optimal regularization parameter (λ.1se) (Figures 4A,B). Stability selection confirmed 5 of the 11 LASSO-selected genes as high-confidence features (Supplementary Table S9). RFE-RF was employed to identify the optimal subset of genes for classification, with the best performance achieved using 10 features. This subset yielded an accuracy of 97.29% and a Cohen’s kappa of 0.9031 under 10-fold cross-validation repeated five times (Figure 4C; Supplementary Table S10). A final Random Forest model constructed using these 10 genes demonstrated strong classification performance and provided interpretable feature importance (Figure 4D; Supplementary Table S11). Notably, all 10 RFE-selected genes were also confirmed as important by the Boruta algorithm, further supporting their relevance (Supplementary Figure S23; Supplementary Table S12). Concurrently, SVM-RFE identified a 30-gene signature that yielded high classification accuracy (up to 98.7%) with minimal error and robust performance (Figure 4E; Supplementary Tables S13, S14). Finally, we selected the intersection of genes screened by the three machine learning methods and determined that the target genes were IRAK3, IL1RN, CD55 and PEA15 (Figure 4F).

To better evaluate the diagnostic performance of the four identified targets for UC, ROC curve analysis was conducted on both the training and validation datasets. The AUC values exceeded 0.90 in the training set and were above 0.80 in the validation set, demonstrating strong discriminatory power (Figures 4I,J). In two independent datasets (GSE87466 and GSE75214), SPI1, along with IRAK3, IL1RN, CD55, and PEA15 showed significantly higher expression in the UC group compared to the normal controls (*P* < 0.05, Figures 4G,H), suggesting that these genes may be upregulated in response to disease-associated regulatory mechanisms. Meanwhile, SPI1 expression showed a correlation with IRAK3, IL1RN, CD55 and PEA15 in human samples (Supplementary Figure S27), supporting a potential regulatory role of SPI1 in promoting downstream target gene expression.

### 3.6 SPI1 regulates macrophage polarization *in Vitro*

**FIGURE 5:**
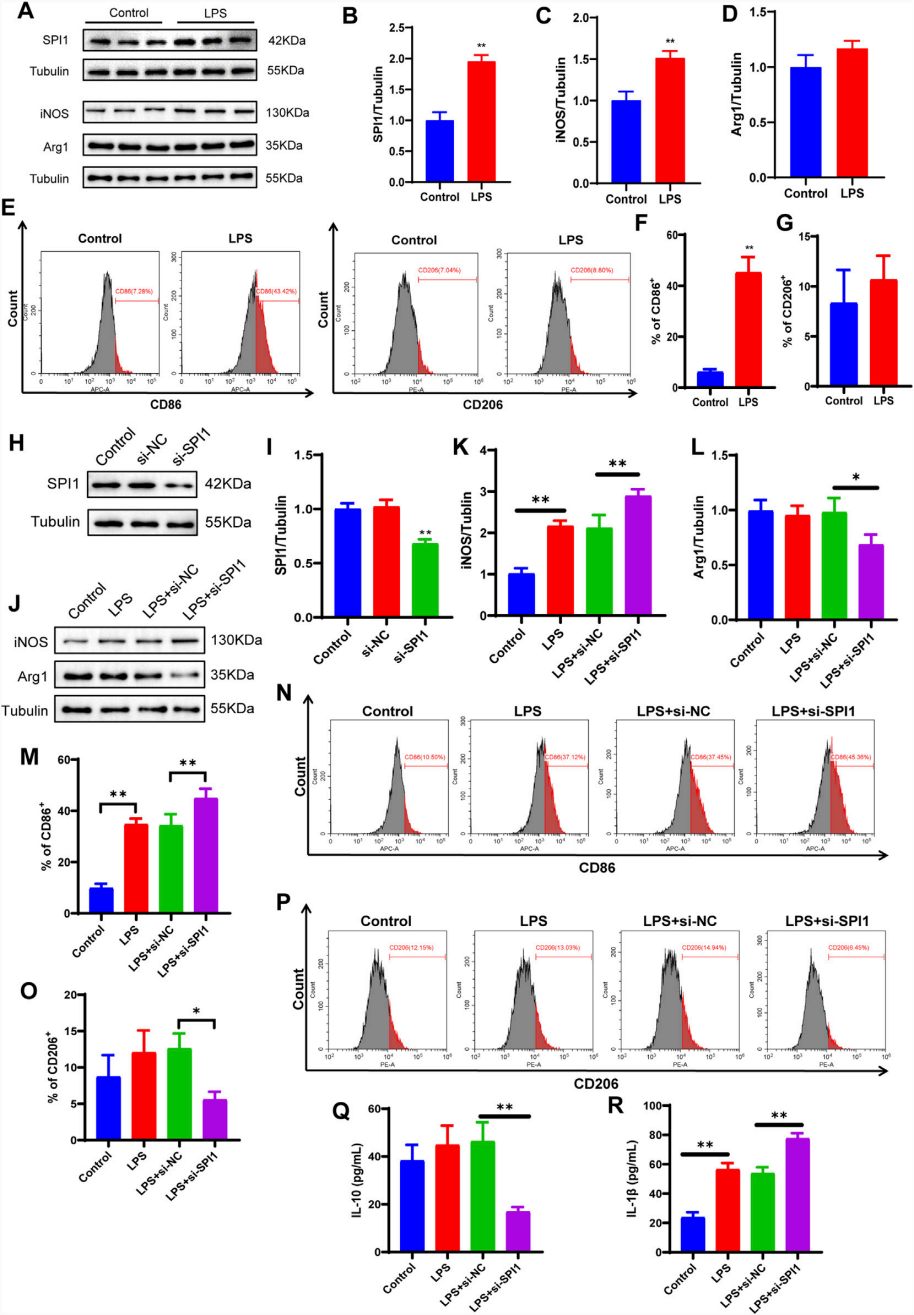
LPS-induced polarization of M1 macrophages is affected by SPI1 in vitro. **(A–D)** The protein expressions of SPI1 **(B)**, iNOS **(C)**, Arg1 **(D)** measured by WB. **(E–G)** Macrophage M1 polarization induced by LPS was detected by flow cytometry. **(H, I)** WB analysis was performed to assess the knockdown efficiency of SPI1 following si-SPI1 treatment, including three groups: untreated control, si-NC (non-targeting siRNA), and si-SPI1. **(J–L)** The expressions of iNOS **(K)** and Arg1 **(L)** measured by WB after SPI1 knockdown. **(M–P)** Macrophage polarization induced by LPS was detected by flow cytometry after SPI1 knockdown. **(Q, R)** Levels of IL-10 **(Q)** and IL-1β **(R)** measured by ELISA after SPI1 knockdown. All experiments were performed in triplicate with independently prepared biological samples. Data are expressed as mean ± SD. *P < 0.05, **P < 0 .01 compared to the other group.

To elucidate the molecular mechanism by which SPI1 regulates macrophage polarization, a series of *in vitro* experiments were conducted. Initially, M1 polarization of RAW264.7 mouse macrophages was successfully induced using LPS. WB analysis confirmed the induction, evidenced by a marked increase in the M1 marker iNOS and a corresponding decrease in the M2 marker Arg1 (*P* < 0.05, Figures 5A,C,D). Flow cytometry further supported this observation, showing a significant upregulation of CD86 in the LPS-treated group, while CD206 levels remained unchanged between groups, reinforcing the effective induction of M1 polarization (*P* < 0.05, Figures 5E–G). Subsequent WB analysis revealed that SPI1 expression was significantly elevated in the LPS group compared to the control (*P* < 0.05, Figures 5A,B). To investigate the functional role of SPI1, we employed siRNA to selectively silence SPI1 expression. The knockdown efficiency was validated through WB (*P* < 0.05, Figures 5H,I). WB analysis also showed that after SPI1 knockout, the level of the M1 marker iNOS was even higher than that induced by LPS, while the M2 marker Arg1 was decreased (*P* < 0.05, Figures 5J-L). ELISA analysis revealed that SPI1 knockdown significantly increased IL-1β secretion while reducing IL-10 levels following LPS stimulation (*P* < 0.05, Figures 5Q,R). Flow cytometry also found that knocking out SPI1 promoted M1 macrophage polarization (Figures 5M-P). These findings suggest that SPI1 plays a complex role in modulating macrophage polarization.

## 4 Discussion

UC is an autoimmune disorder characterized by aberrant activation of inflammatory responses. Impaired macrophage function is closely linked to the underlying inflammatory mechanisms of UC. During the active phase of UC, an imbalance in macrophage subsets has been reported, potentially driven by alterations in their polarization states (Du et al., 2023). An increase in pro-inflammatory M1 macrophages is commonly observed, accompanied by a reduction in anti-inflammatory M2 macrophages. Notably, inducing macrophage polarization toward the M2 phenotype can mitigate oxidative stress in epithelial cells and substantially reduce UC-associated intestinal damage.

To further investigate macrophage dynamics in UC, we analyzed scRNA-seq from UC patients. This analysis revealed a significant increase in macrophage abundance, underscoring their central role in UC pathogenesis. We then employed the pySCENIC algorithm to reconstruct gene regulatory networks based on co-expression patterns and transcription factor binding predictions. Regulon analysis highlighted significant upregulation of macrophage activation pathways in UC, with SPI1 identified as a highly active transcription factor.

SPI1 (PU.1) is an essential regulator of macrophage immunity and belongs to the ETS transcription factor family (Lloberas et al., 1999; Zakrzewska et al., 2010). The scRNA-seq analysis demonstrated strong enrichment of SPI1 and its target genes in macrophage activation pathways among UC patients, consistent with SPI1’s important role in regulating macrophage function during UC pathogenesis. These findings align with previous reports indicating elevated SPI1 activity in IBD (Huang et al., 2022; Nowak et al., 2022). Studies have shown that increased SPI1 expression could regulate microglial immune activity and attenuate inflammation near amyloid plaques in Alzheimer’s disease (Kim et al., 2024), suggesting its broader role in restraining aberrant immune responses. *In vitro* experiments further support SPI1’s immunoregulatory function. Our findings indicate that upon LPS stimulation, macrophages that have polarized toward the M1 phenotype exhibit a significant increase in SPI1 expression. However, SPI1 knockout significantly enhanced the expression of pro-inflammatory markers such as IL-1β, while reducing levels of the anti-inflammatory cytokine IL-10. This shift indicates a heightened M1 polarization in the absence of SPI1, highlighting its role in maintaining macrophage functional balance. Collectively, although SPI1 expression increases during M1 polarization, our study suggested that SPI1 may serve to restrain excessive M1 activation and maintain macrophage homeostasis. In response to inflammatory stimuli such as LPS, heightened SPI1 expression likely represents an adaptive mechanism. This mechanism helps prevent detrimental overactivation of M1 macrophages and facilitates their transition to the anti-inflammatory M2 state. In addition, SPI1 boosts phagocytosis in microglia and macrophages, which helps alleviate ongoing neuroinflammation following cerebral hemorrhage (Zhang et al., 2024). Conversely, SPI1 knockdown under LPS exposure can impair phagocytosis, potentially exacerbating and prolonging inflammatory responses.

To further investigate the regulatory role of SPI1 in macrophages, we integrated SPI1-associated downstream gene regulatory networks with microarray sequencing data. Using machine learning algorithms, we identified and validated IRAK3, IL1RN, CD55 and PEA15 as critical downstream targets of SPI1. In UC patients, the expression levels of these genes were significantly elevated compared to healthy controls.

IRAK3, or interleukin-1 receptor-associated kinase 3, is a key negative regulator of the Toll-like receptor (TLR) signaling pathway. It is mainly expressed in monocytes and macrophages. In these cells, IRAK3 plays an important role in preventing excessive activation of the innate immune system and helps maintain immune balance (Wesche et al., 1999; Tunali et al., 2023). In mouse models of DSS-induced colitis, deletion of IRAK3 significantly lowers serum IL-1β levels and reduces intestinal inflammation (Berglund et al., 2010). In patients with U, IRAK3 expression in the intestinal mucosa rises during active disease but returns to near-normal levels during remission (Günaltay et al., 2014). This pattern suggests that IRAK3 may help regulate immune responses during flare-ups of the disease. Recent studies also link IRAK3 to macrophage polarization. IRAK3 expression is closely associated with the shift toward the M2 macrophage phenotype (Chinju et al., 2022). This shift may represent a compensatory response, where the body promotes M2 polarization to limit inflammation and support tissue repair.

IL1RN encodes the interleukin-1 receptor antagonist (IL-1RA), an important regulator of inflammation. IL-1 R A blocks the binding of IL-1 to its receptor. In doing so, it helps suppress downstream inflammatory signals (Zou et al., 2021). IL1RN is known for its protective role in many autoimmune diseases. In a mouse model of rheumatoid arthritis, loss of IL1RN leads to stronger and more persistent inflammation (Horai et al., 2000). IL1RN expression often increases in UC. This upregulation may help reduce IL-1β production and provide a negative feedback loop to control mucosal inflammation (Ashwood et al., 2004). Recent studies add another layer to its function. IL1RN may also support the shift of macrophages toward the anti-inflammatory M2 phenotype. This effect could help protect intestinal tissue and limit injury (Cao et al., 2023).

CD55, also known as decay-accelerating factor, is a crucial regulator of the complement system (An et al., 2023). In the context of UC, CD55 expression is upregulated in response to inflammation. Notably, its expression levels show a positive correlation with the severity of mucosal inflammation (Inaba et al., 1998). This increase likely reflects a compensatory response aimed at limiting excessive immune activation. Supporting this, CD55-deficient mice exhibit aggravated colitis symptoms (Lin et al., 2004). Mechanistically, CD55 exerts anti-inflammatory effects by modulating autocrine C3aR1 and C5aR1 signaling. It promotes the polarization of dendritic cells toward a tolerogenic M2 phenotype (Strainic et al., 2019) and inhibits complement convertase activity, thereby reducing macrophage infiltration and tissue injury (Hsu et al., 2025).

In this study, we found that PEA15 was markedly upregulated in the intestinal tissues of patients with UC. PEA15, a phosphoprotein involved in regulating the ERK/RSK2 signaling pathway, has been shown to suppress cell proliferation and migration across various cellular contexts (Krueger et al., 2005; Gawecka et al., 2012). Accumulating evidence also implicates PEA15 in the modulation of inflammatory responses. It could attenuate LPS-induced oxidative stress and DNA damage, exerting notable anti-inflammatory effects. Additionally, PEA15 inhibits the MARK signaling pathway, thereby reducing inflammation triggered by external stimuli (Yong et al., 2018). These findings suggest that elevated PEA15 expression may represent a compensatory feedback mechanism in response to an inflammatory microenvironment. Notably, exosomes derived from M2-polarized macrophages in the intestinal mucosa of IBD patients have been shown to interact with PEA15, indicating a potential link between PEA15 upregulation and macrophage polarization status (Liu et al., 2025).

This study utilized public scRNA-seq and microarray data lacking key clinical parameters such as age and gender beyond basic disease status. Since these factors significantly influence UC pathogenesis, their absence may contribute to heterogeneity in immune cell composition, gene expression, and regulatory networks (Tedde et al., 2008; Chhibba et al., 2024). Although standardization and batch correction were applied during scRNA-seq analysis and WGCNA module construction to minimize non-biological variation, residual bias from missing clinical features cannot be excluded. Consequently, these findings necessitate validation and expansion using larger cohorts with comprehensive clinical annotation. It should be noted that the *in vitro* model is inherently deficient in its representation of the inflammatory microenvironment in UC, where dynamic intercellular crosstalk and multiple signaling pathways collectively regulate the functional states and polarization of macrophages. Consequently, the establishment of suitable animal models for *in vivo* validation is essential to assess the pathophysiological relevance of SPI1-mediated macrophage polarization. While our study demonstrates SPI1’s overall influence on macrophage polarization, further experimental validation is required to elucidate the functional roles and regulatory relationships of its candidate downstream targets, IRAK3, IL1RN, CD55 and PEA15. Moreover, the lack of *in vivo* human studies limits the direct clinical translatability of our findings, underscoring the need for future validation in patient-derived tissue samples or *ex vivo* systems that better recapitulate human disease physiology.

## 5 Conclusion

In this study, we identified the transcription factor SPI1 related to the polarization of UC macrophages in scRNA-seq data, and validated its regulatory role in macrophage polarization using *in vitro* experiments. Furthermore, potential downstream targets of SPI1—IRAK3, IL1RN, CD55 and PEA15—were identified through WGCNA and machine learning approaches based on microarray data. These findings offer novel insights into the regulatory mechanisms by which SPI1 influences M1 polarization in UC macrophages and may facilitate the development of personalized therapeutic strategies for UC patients.

## Data Availability

The datasets presented in this study can be found in online repositories. The names of the repository/repositories and accession number(s) can be found below: http://www.ncbi.nlm.nih.gov/geo.

## Abbreviations

AUC, Area Under the Curve; AUCell, Area Under the Curve Cell-level scoring; CAC, Colitis-Associated Cancer; CSI, Cell-Specific Index; CV, coefficient of variation; DE, differential expression; EMT, epithelial-to-mesenchymal transition; GEO, Gene Expression Omnibus; GRN, Gene regulatory networks; GS, gene significance; GSEA, Gene Set Enrichment Analysis; IBD, Inflammatory Bowel Disease; IECs, intestinal epithelial cells; IL-1RA, interleukin-1 receptor antagonist; JSD, Jensen-Shanno Divergence; MAD, median absolute deviation; MM, module membership; ntree, number of decision trees; rasMat, regulon activity score matrix; RFE-RF, Recursive feature elimination with a random forest classifier; ROC, receiver operating characteristic; RSS, Regulon Specificity Score; scRNA-seq, Single-cell RNA Sequencing; SD, standard deviation; siRNA, small interfering RNA; SVM-RFE, Support Vector Machine-Recursive Feature Elimination; TLR, Toll-like receptor.
