# Supplementary material for: Integrative analysis of single-cell and microarray data reveals SPI1-centered macrophage regulatory signatures in ulcerative colitis
Source: Front Genet. 2025 Sep 5;16:1617834. doi: 10.3389/fgene.2025.1617834 (PMC12446007; doi:10.3389/fgene.2025.1617834)
Supplement: Supplementary file 1 [file Supplementaryfile1.docx]

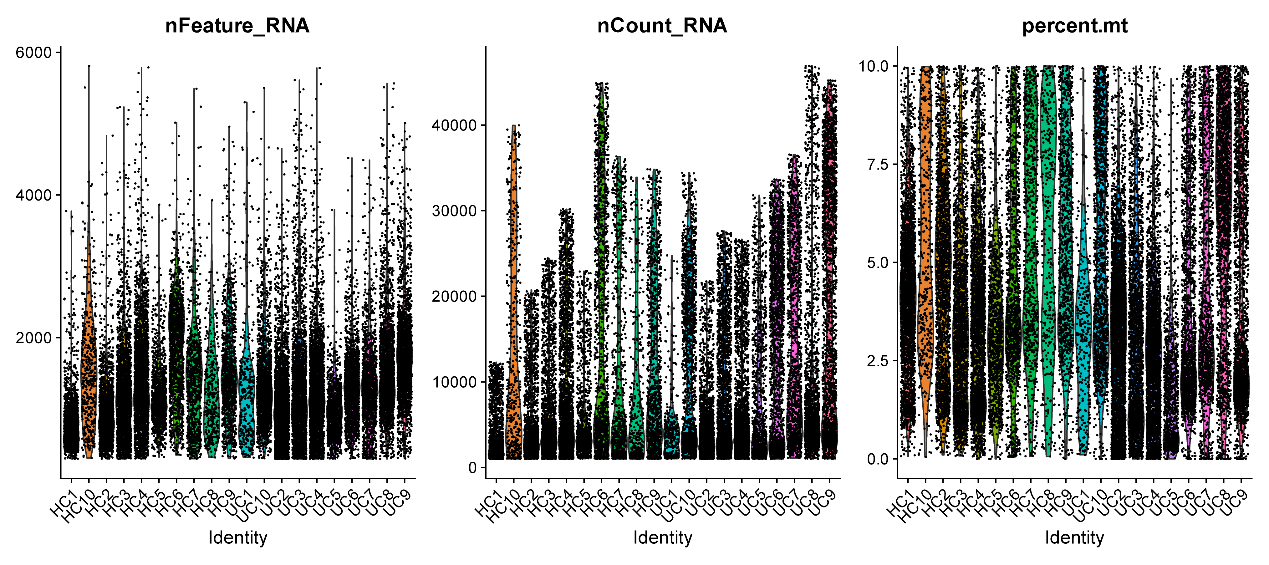


Figure S1 ScRNA-seq data underwent quality control filtering to retain cells with 300-6,000 detected genes, <10% mitochondrial reads, >1,000 UMIs, and total counts below the 97th percentile.


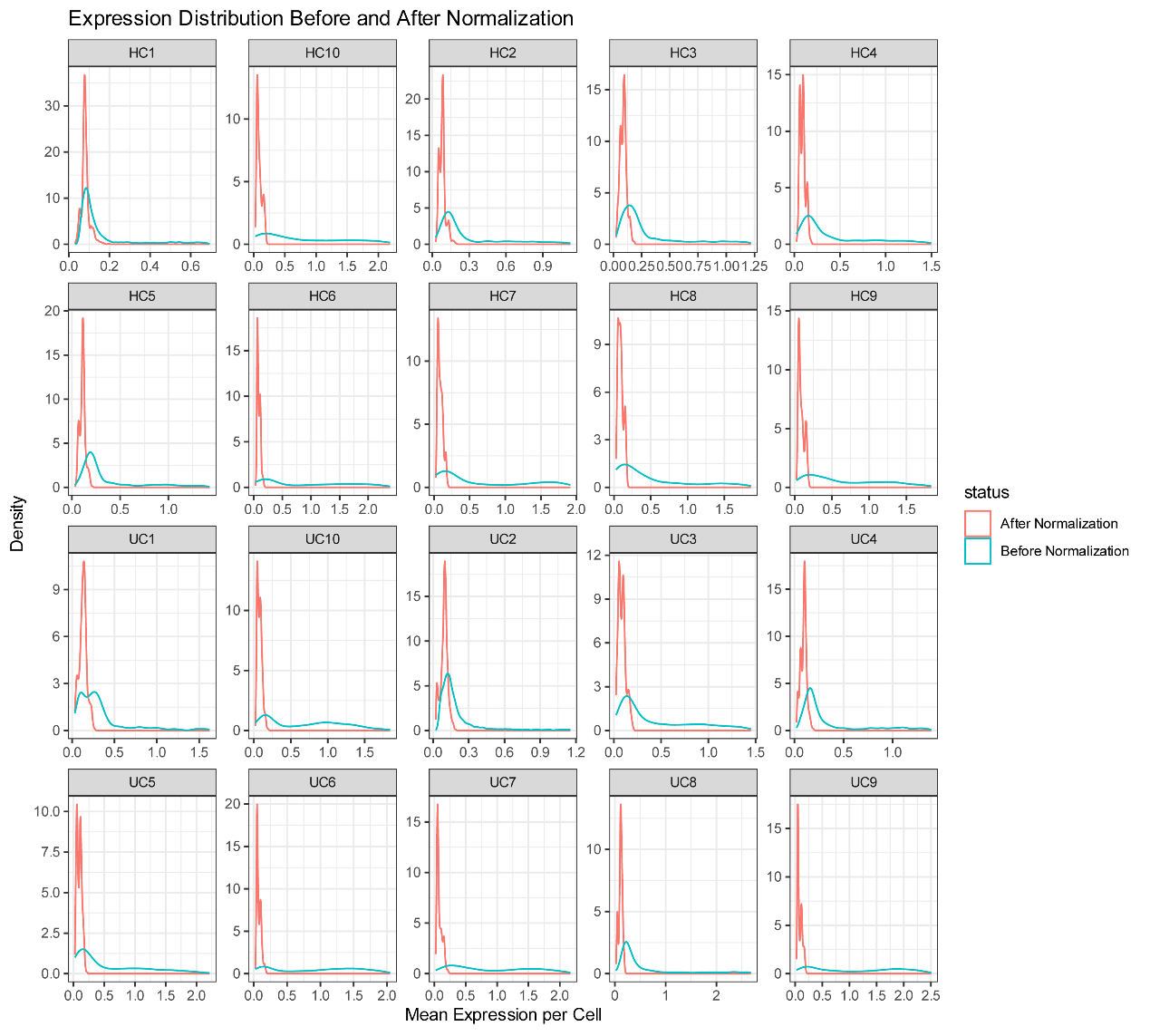


Figure S2 Normalization of ScRNA-seq


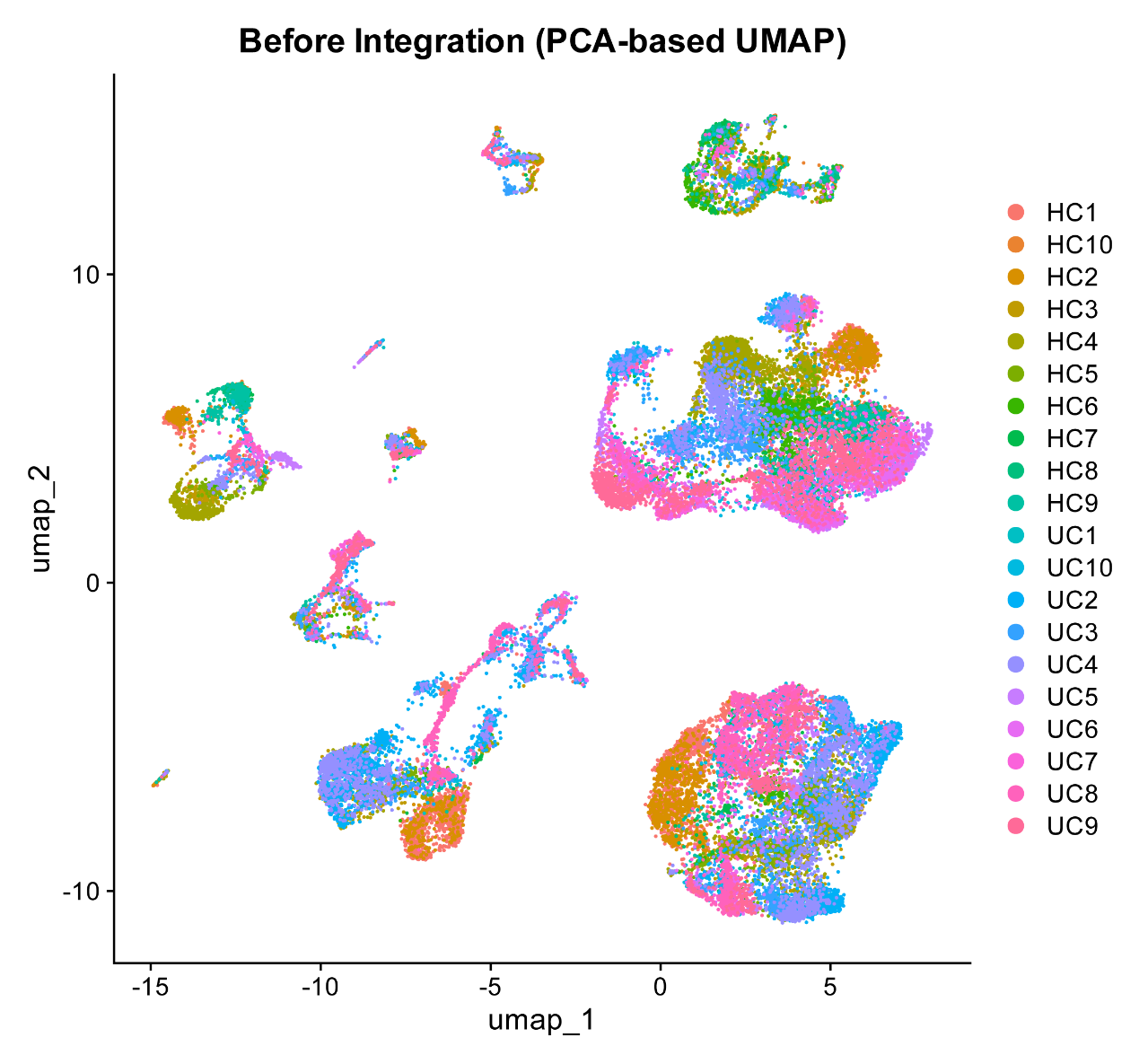


Figure S3 PCA-based UMAP before integration in scRNA-seq.


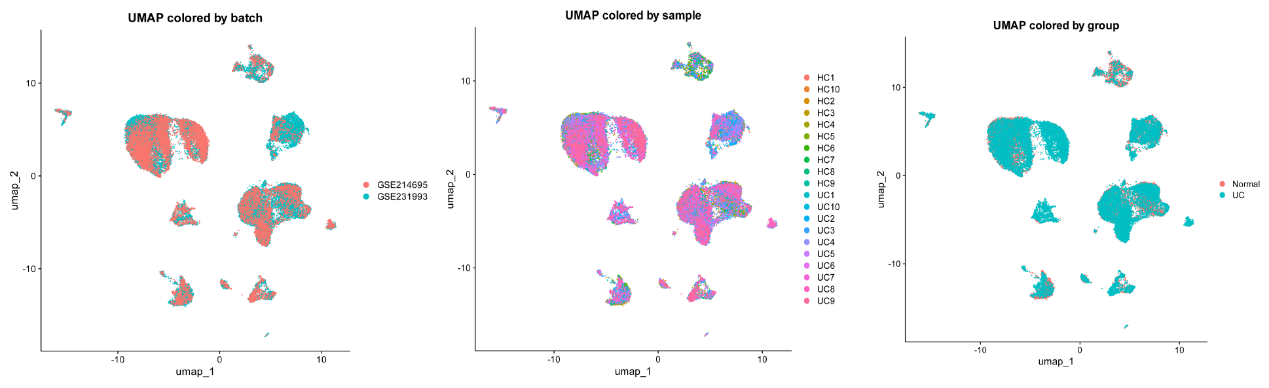


Figure S4 UMAP after harmony in scRNA-seq.


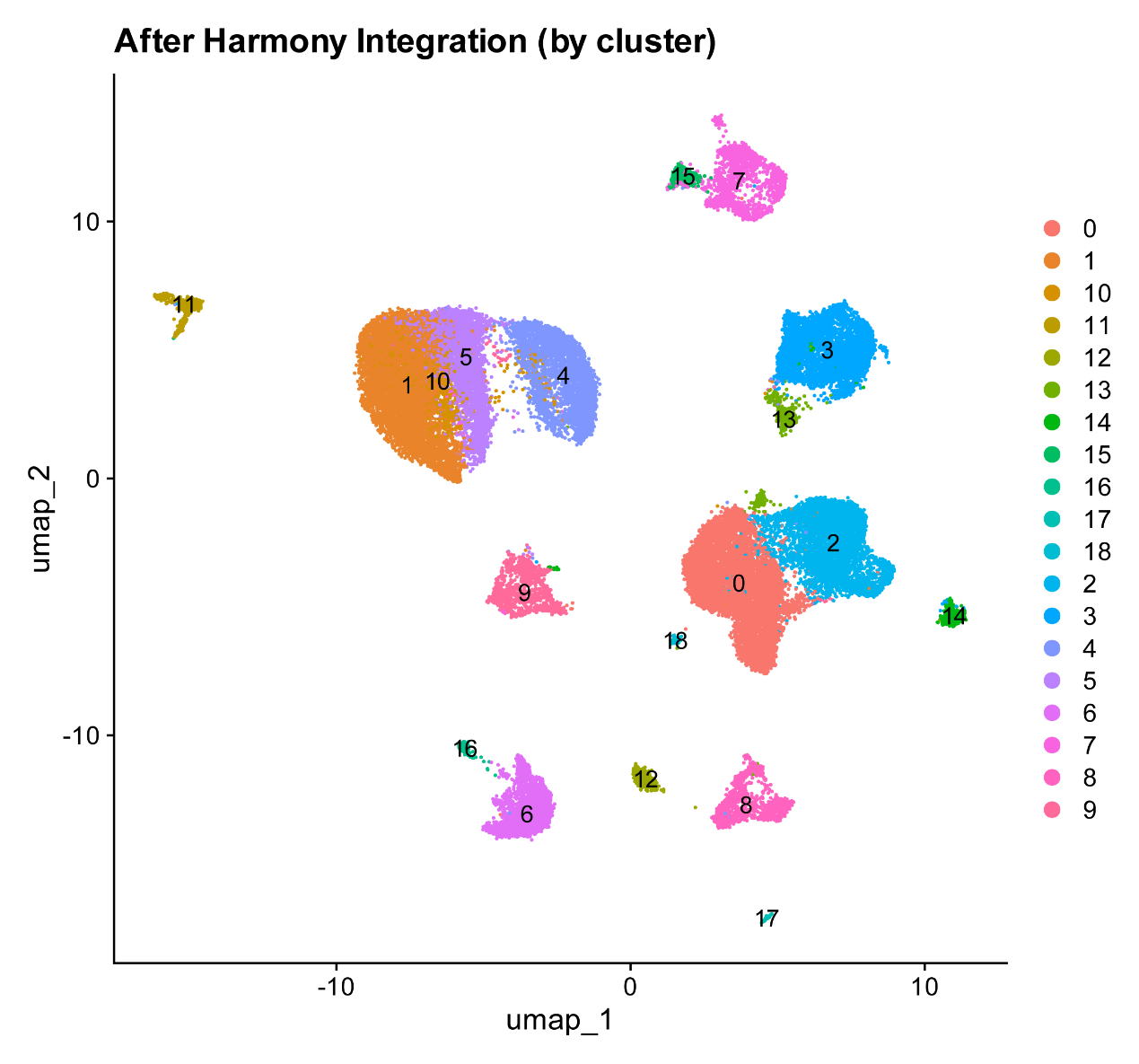


Figure S5 An SNN graph was constructed based on the top 40 harmony dimensions, and Louvain clustering at 0.4 resolution yielded 18 distinct cell clusters


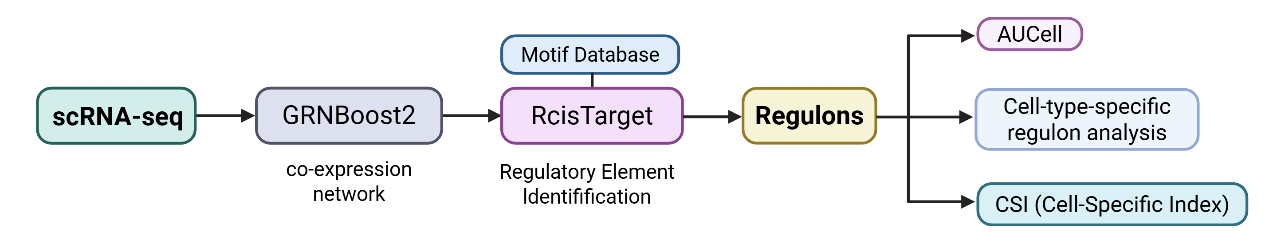


Figure S6 Workflow of pySCENIC


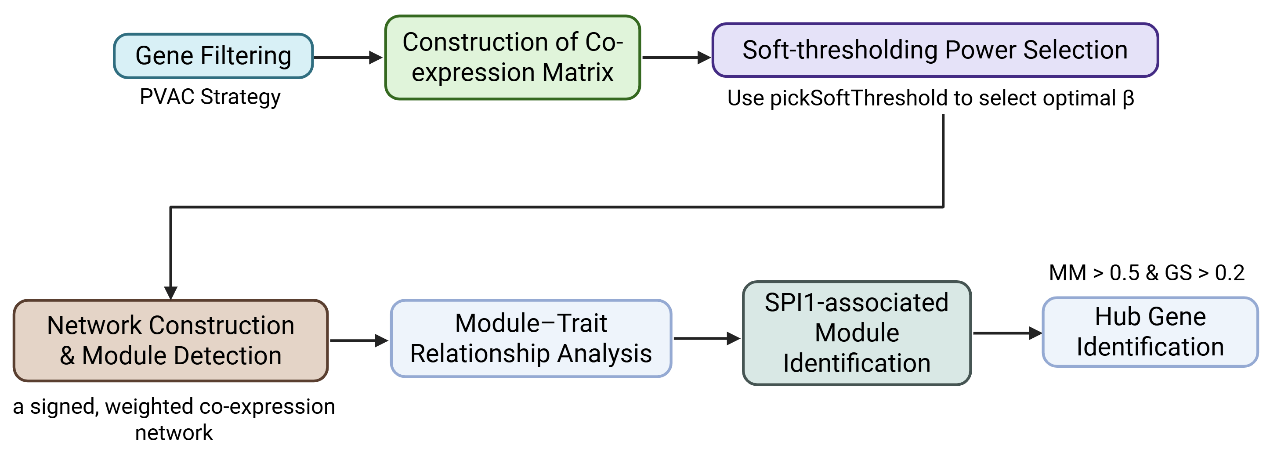


Figure S7 Workflow of WGCNA


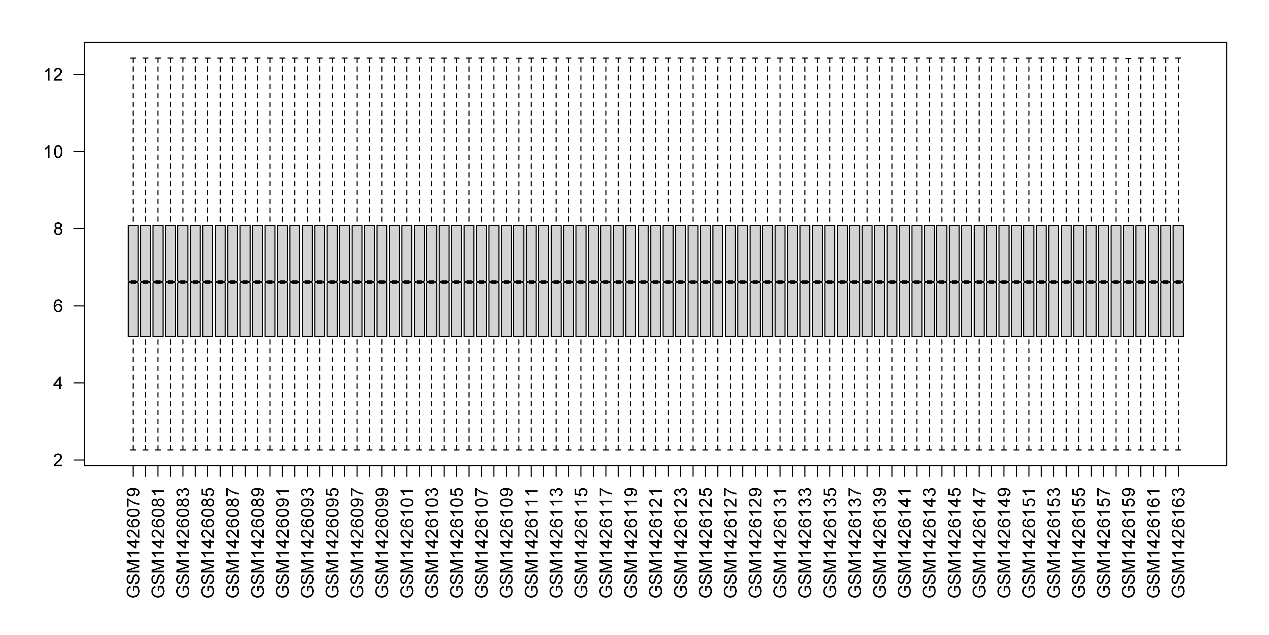


Figure S8 GSE75214 dataset after normalization preprocessing for differential expression analysis.


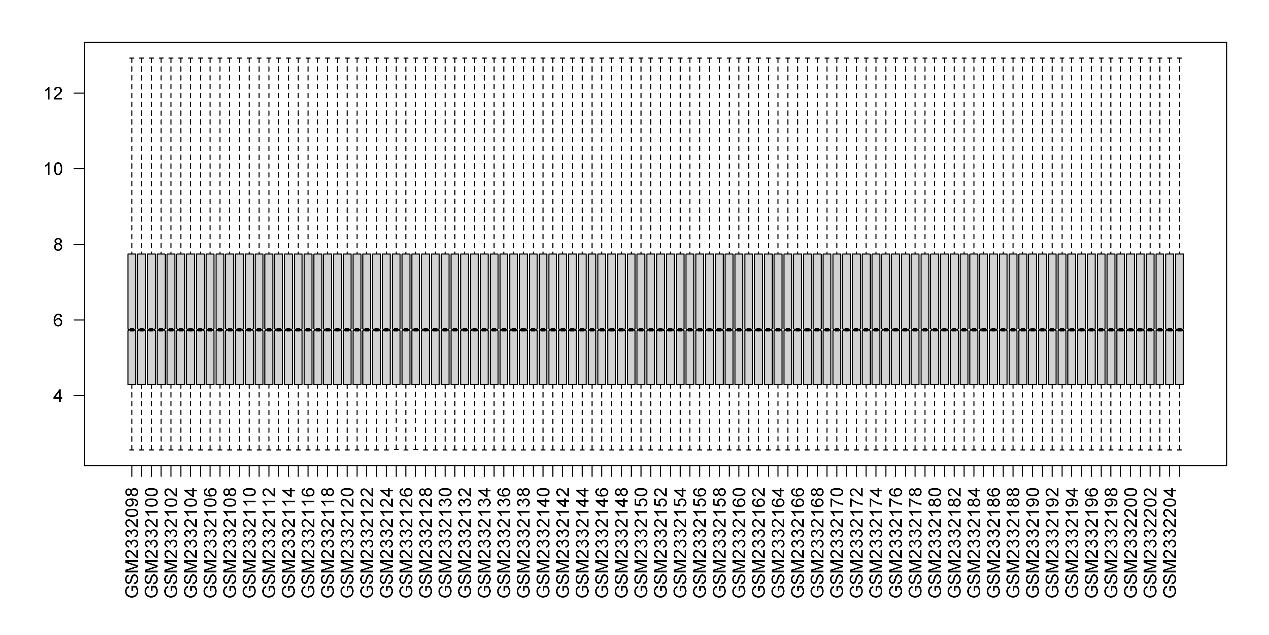


Figure S9 GSE87466 dataset after normalization preprocessing for limma differential expression analysis.


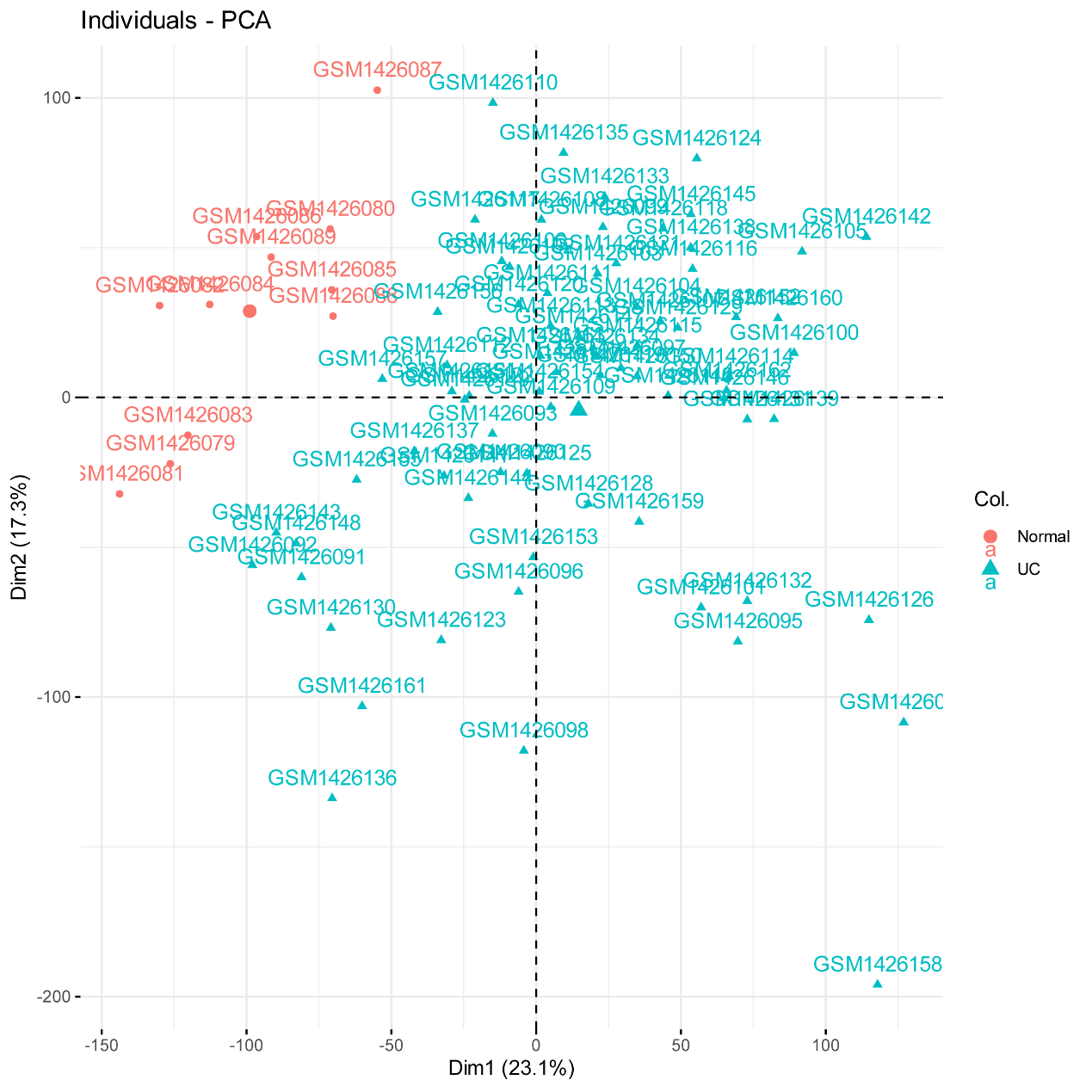


Figure S10 PCA-based Anomaly Detection Results in GSE75214 dataset.


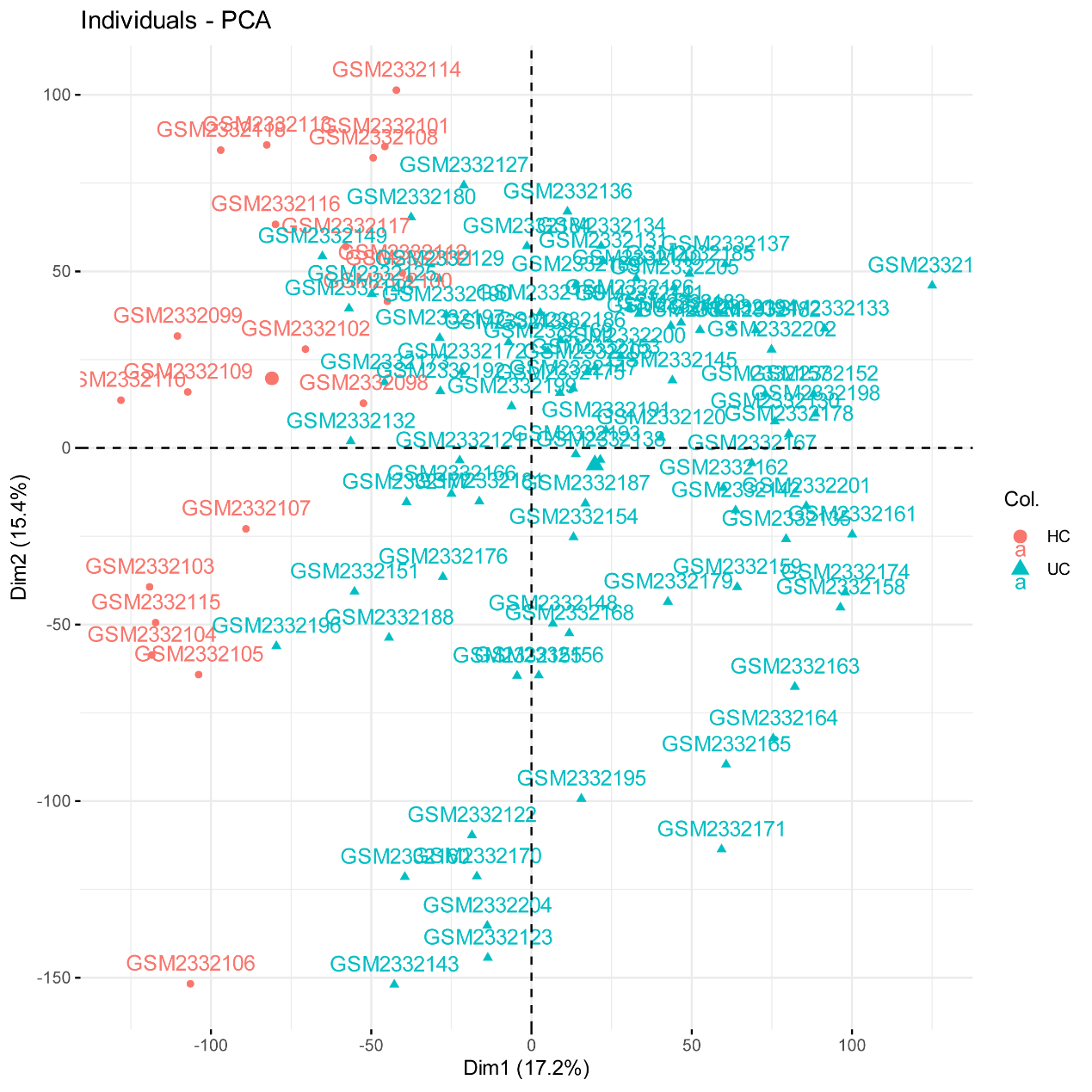


Figure S11 PCA-based Anomaly Detection Results in GSE887466 dataset.


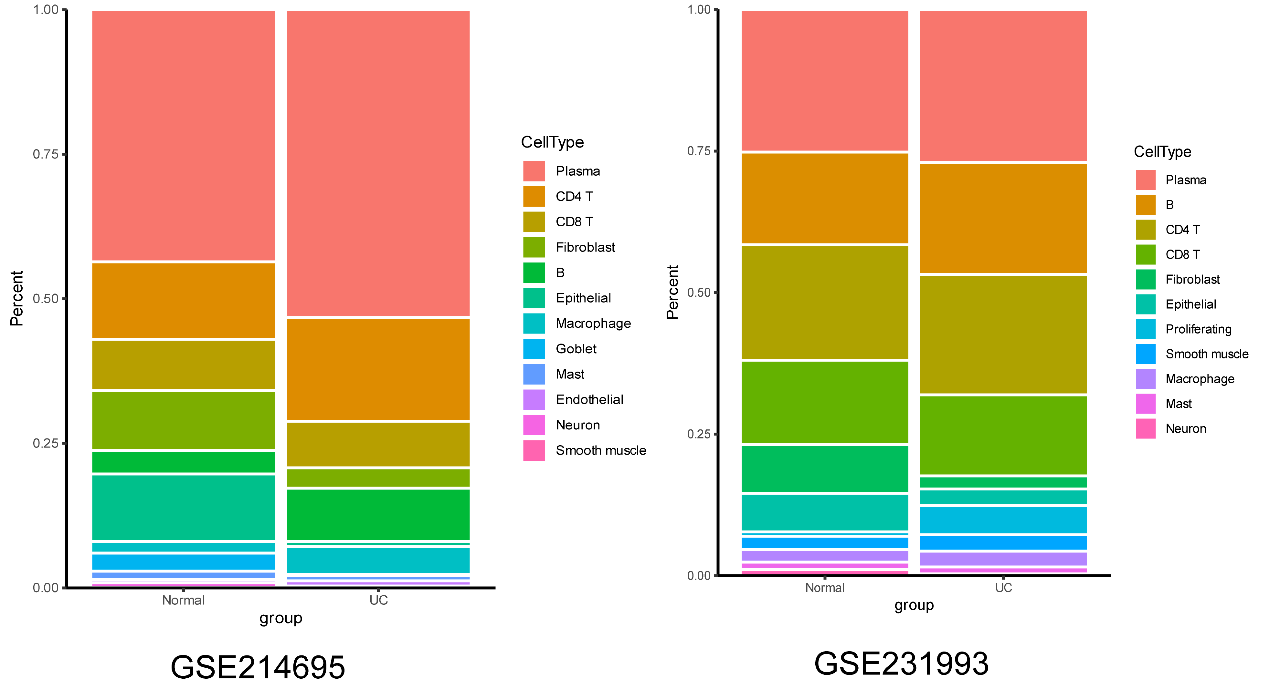


Figure S12 Comparison of cellular composition between ulcerative colitis (UC) and normal control groups across datasets GSE214695 and GSE231993 respectively.


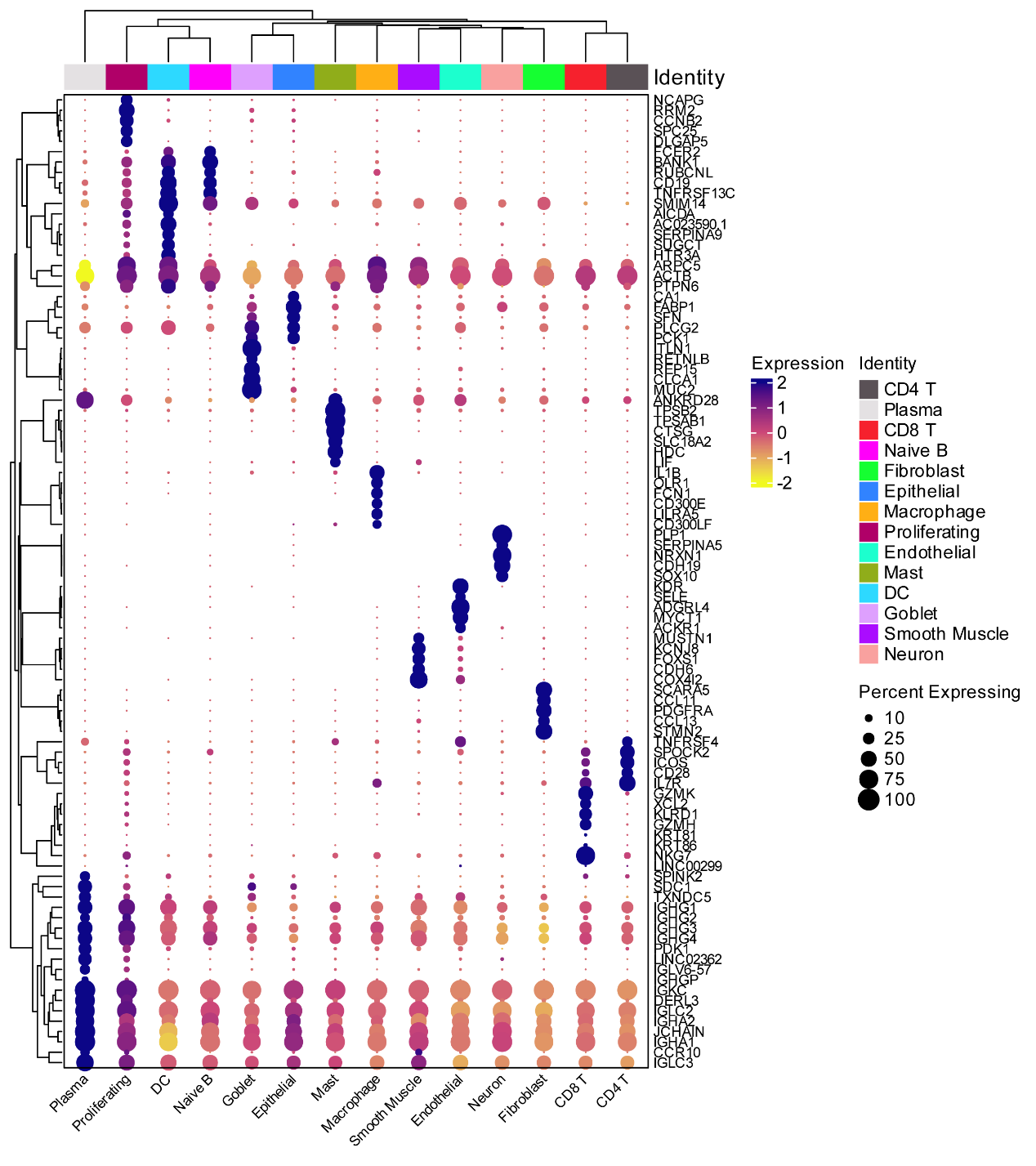


Figure S13 Dot plot heatmap showing gene expression profiles across the different cell types.


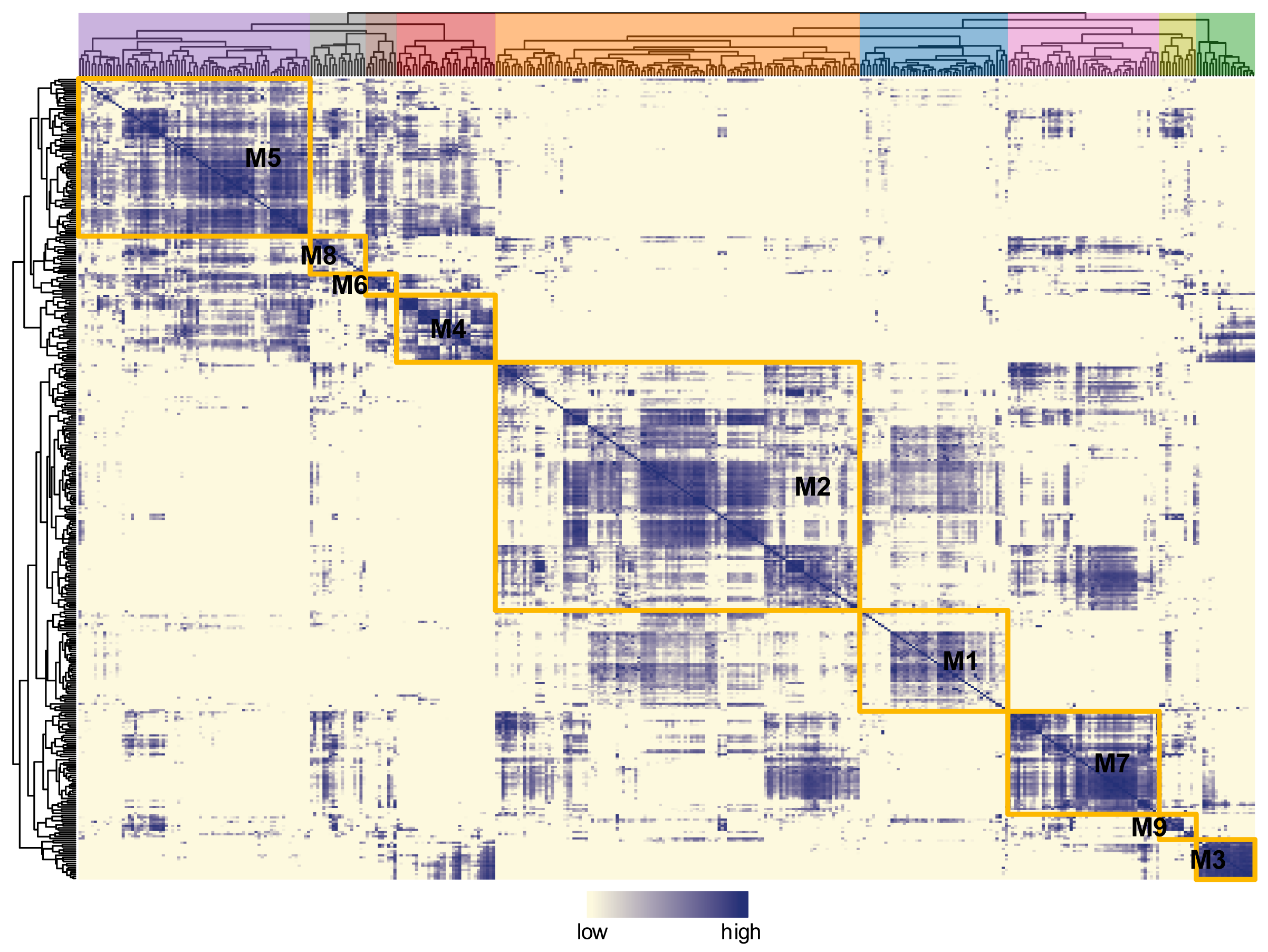


Figure S14 Identified regulon modules based on the regulon CSI matrix.


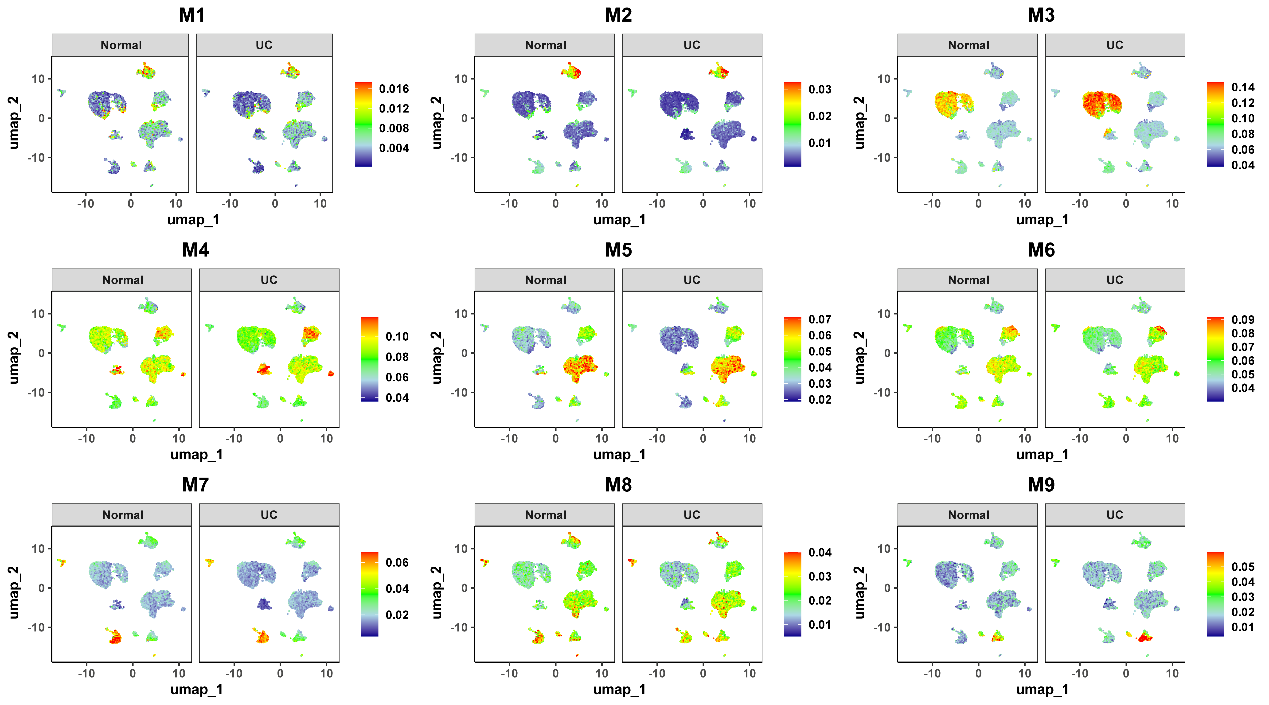


Figure S15 The average activity of regulons within each module.


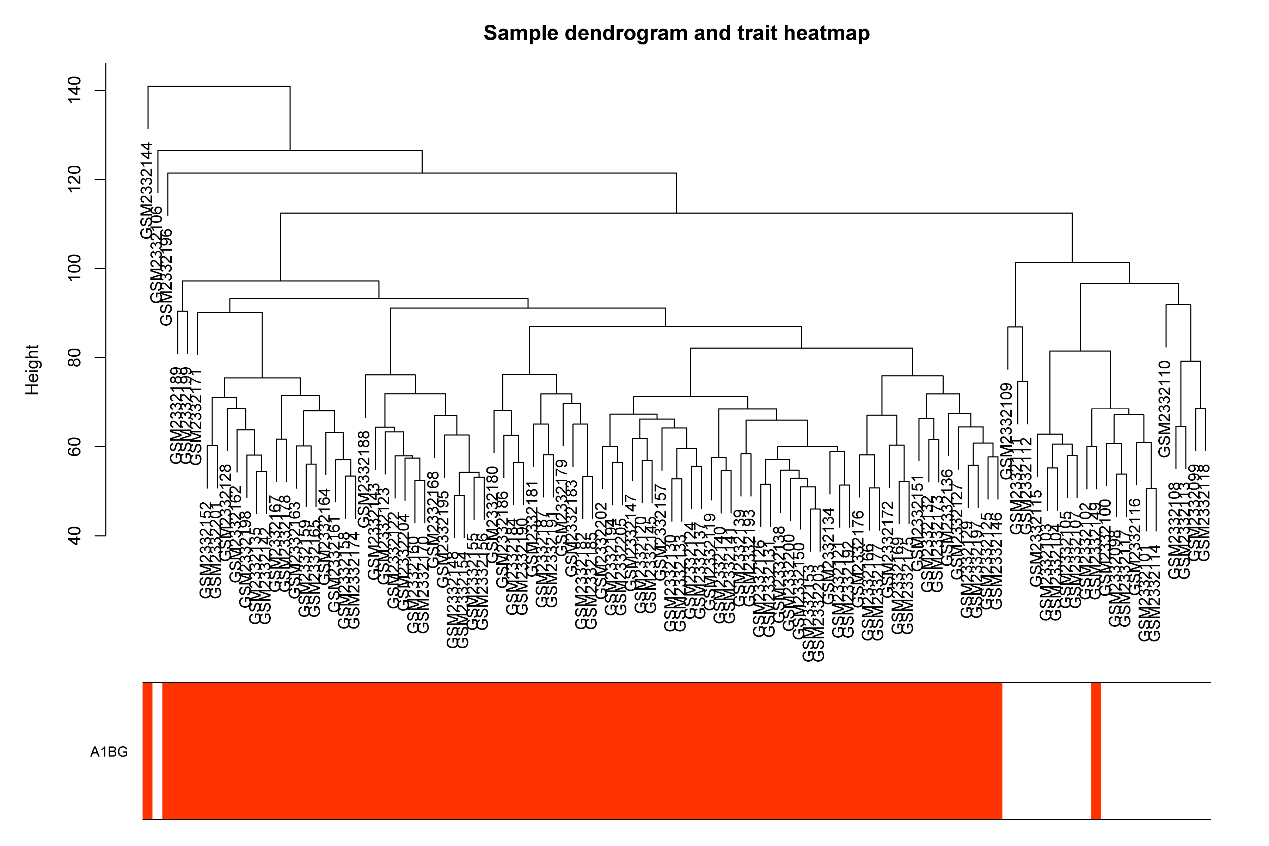


Figure S16 Sample dendrogram and trait expression heatmap in GSE87466 dataset.


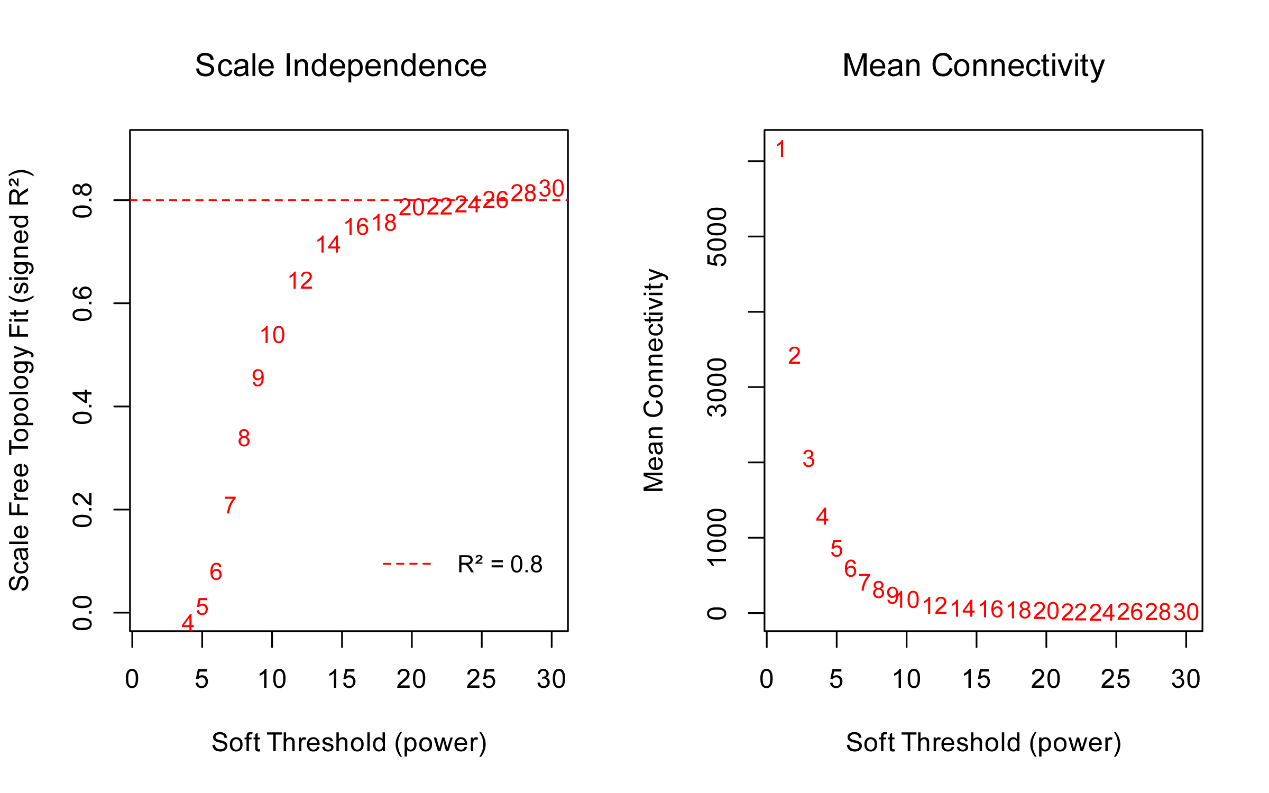


Figure S17 A soft-threshold power of β = 14 was selected to construct a scale-free network in GSE87466.


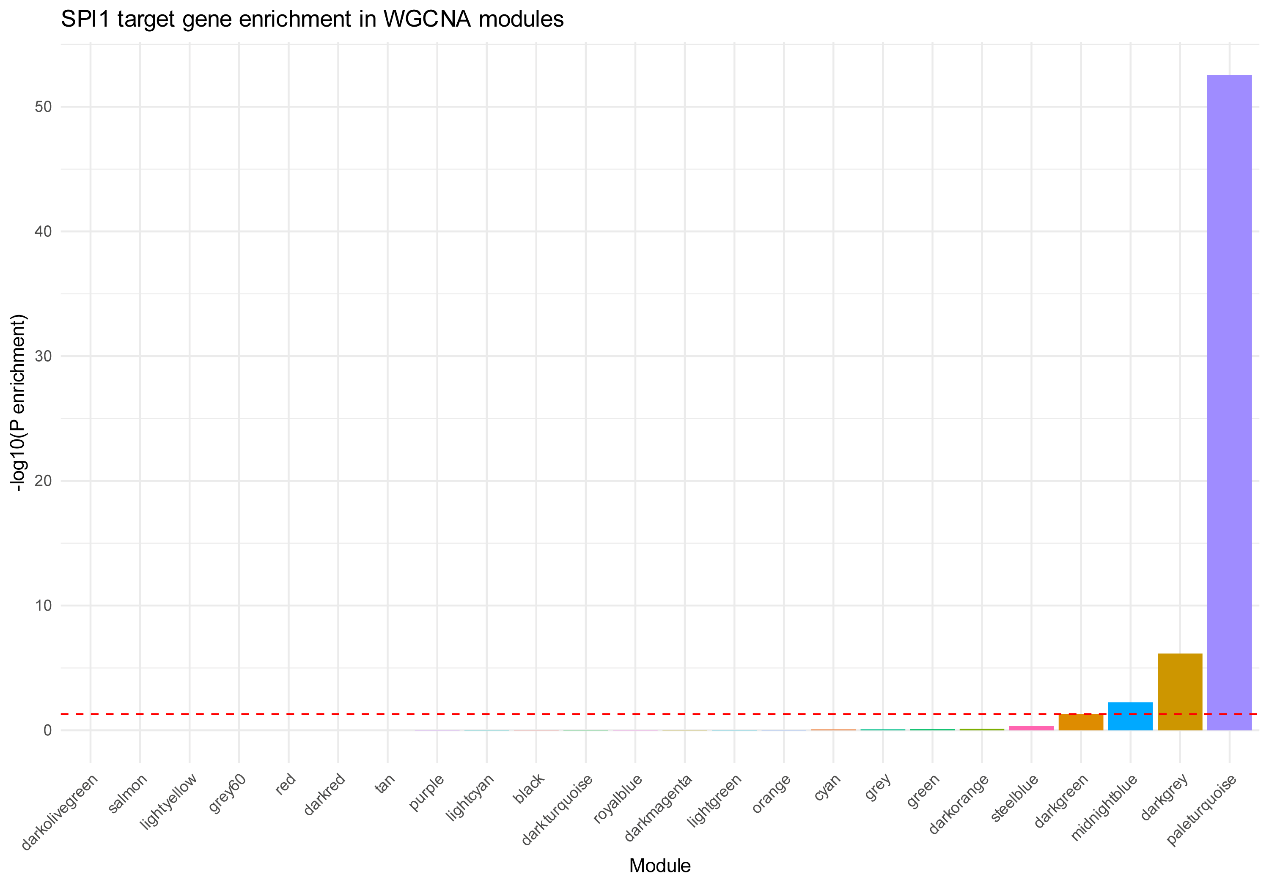


Figure S18 The MEpaleturquoise module showed significant enrichment of SPI1 regulon genes in GSE87466.


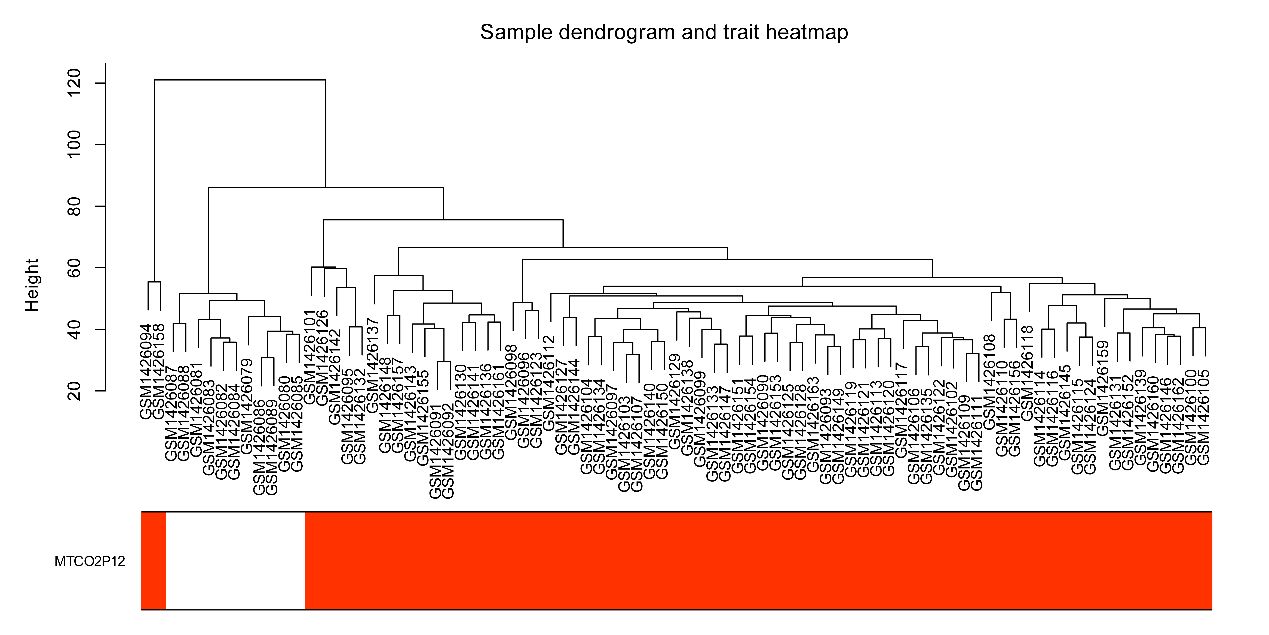


Figure S19 Sample dendrogram and trait expression heatmap in GSE75214 dataset.


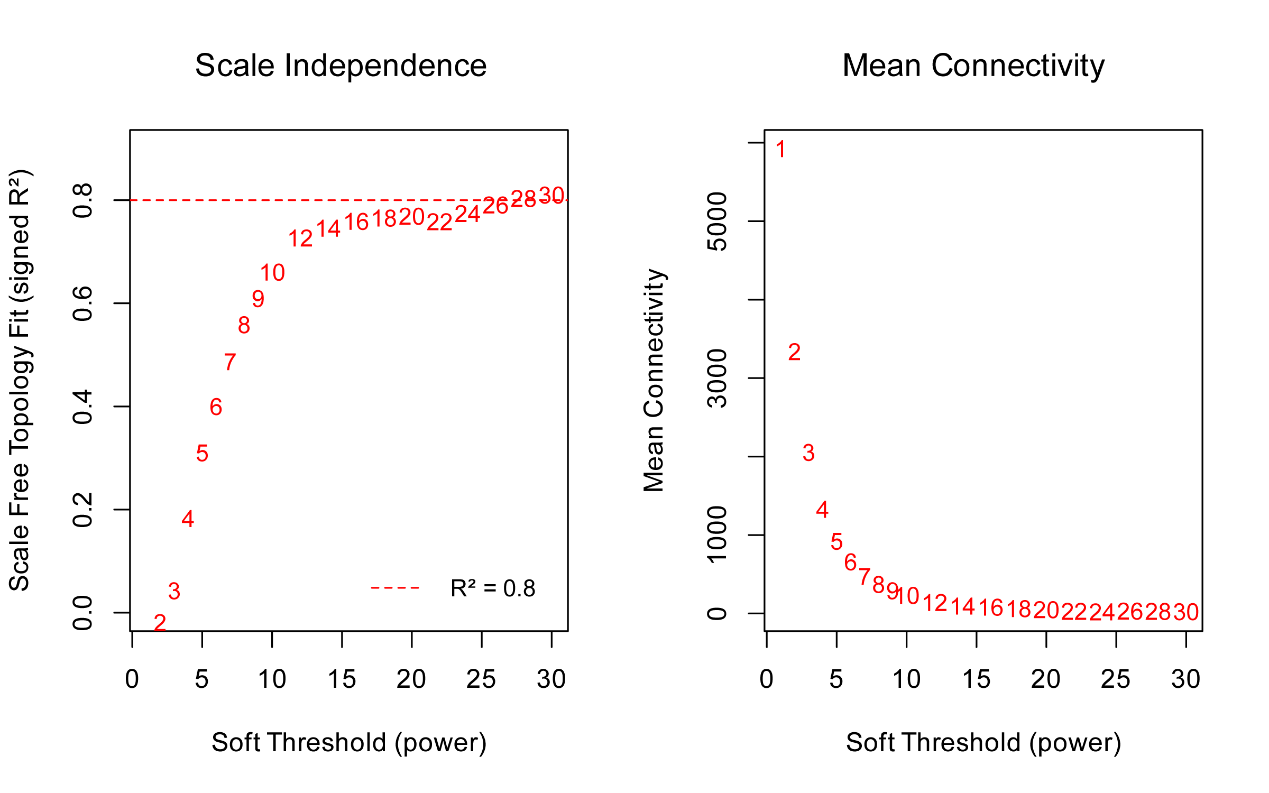


Figure S20 A soft-threshold power of β = 12 was selected to construct a scale-free network in GSE75214.


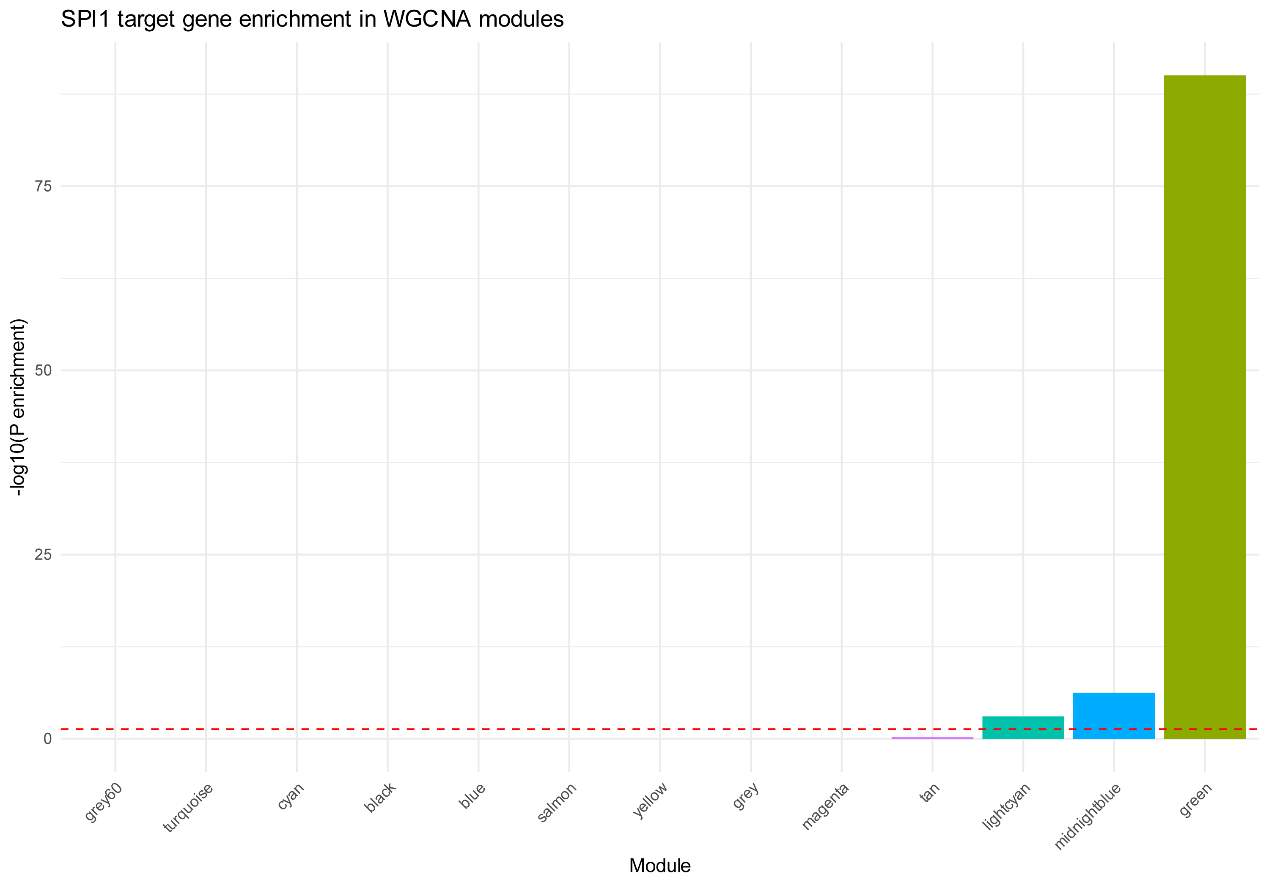


Figure S21 The MEpaleturquoise module showed significant enrichment of SPI1 regulon genes in GSE87466.


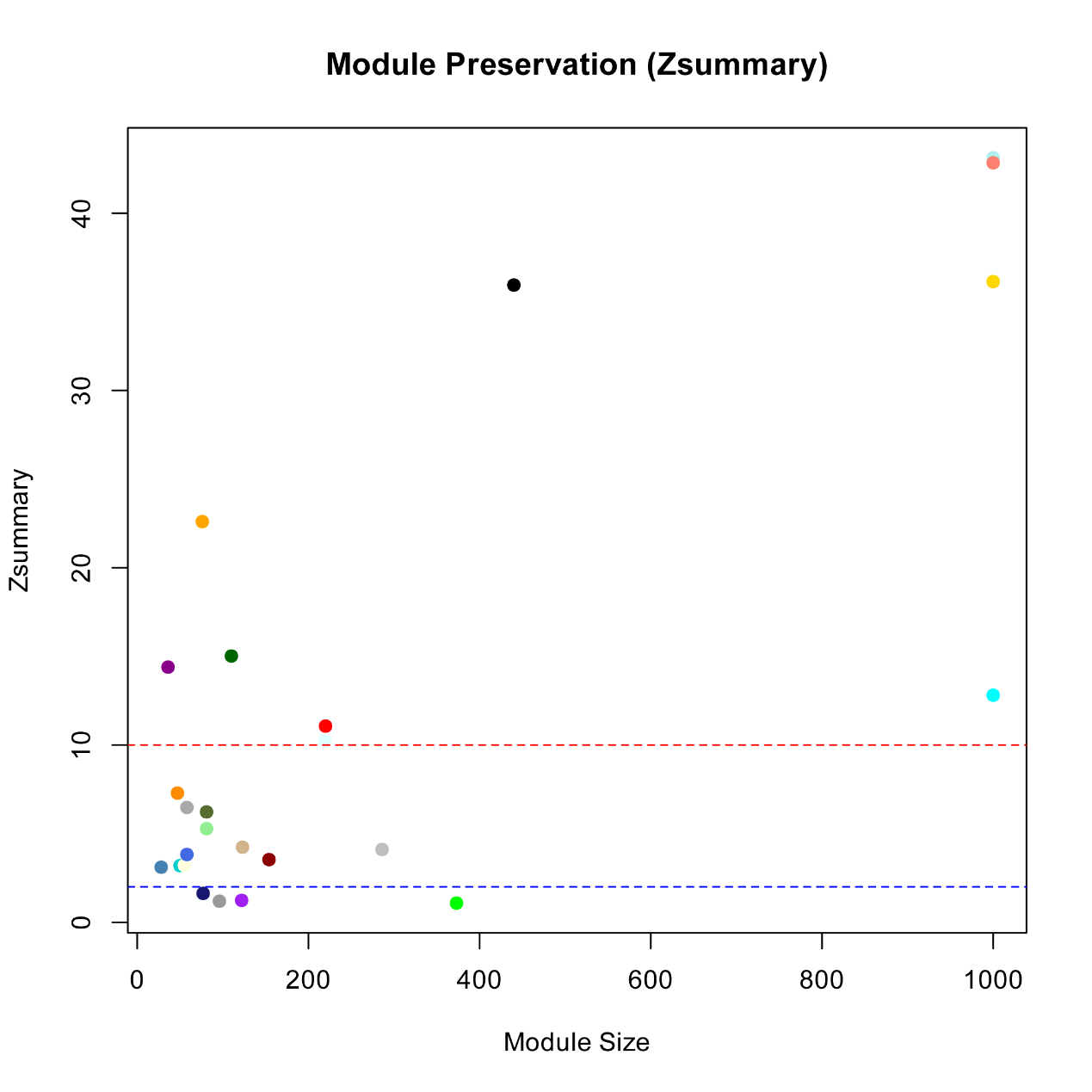


Figure S22 Module preservation analysis between GSE87466 and GSE75214. Most modules showed Zsummary > 2, indicating moderate to strong preservation of gene co-expression patterns across datasets.


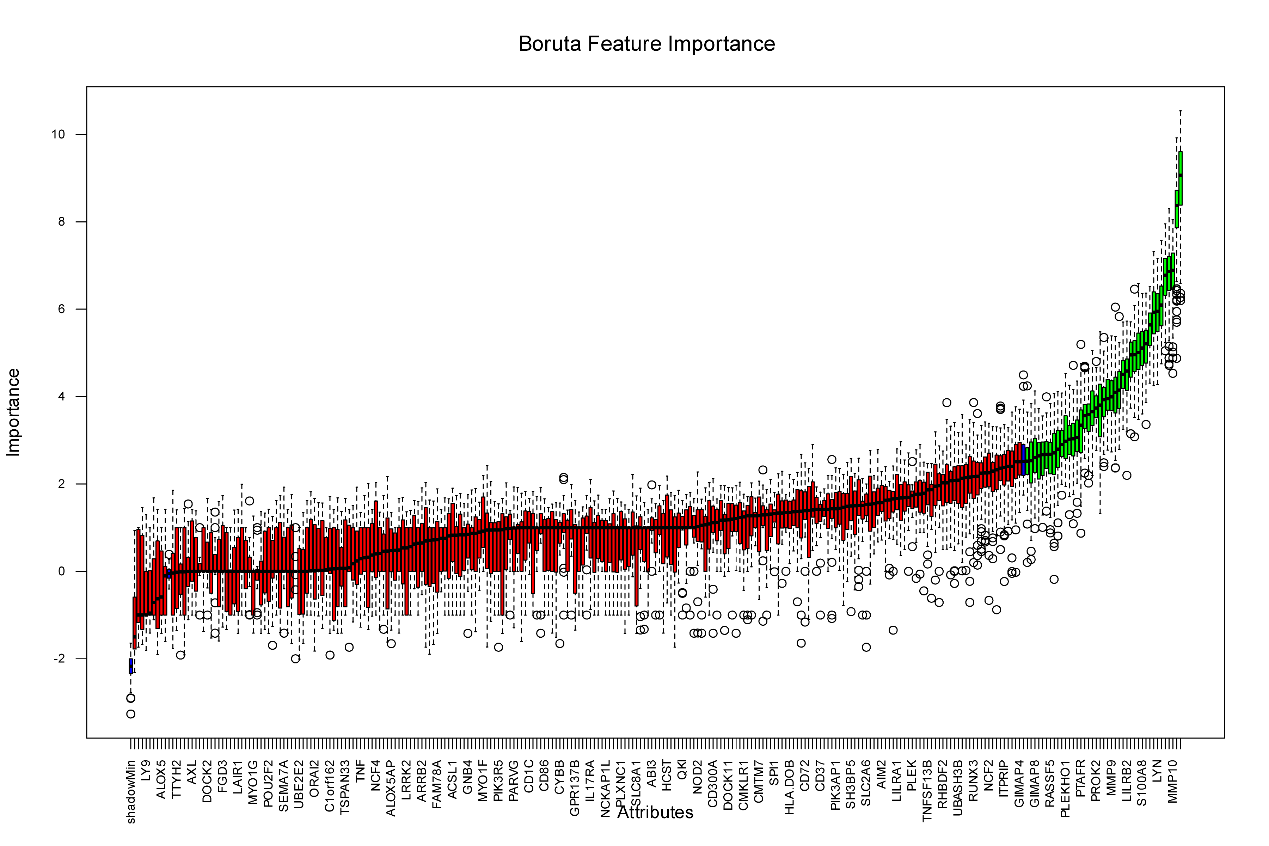


Figure S23 Boruta-based feature importance ranking.


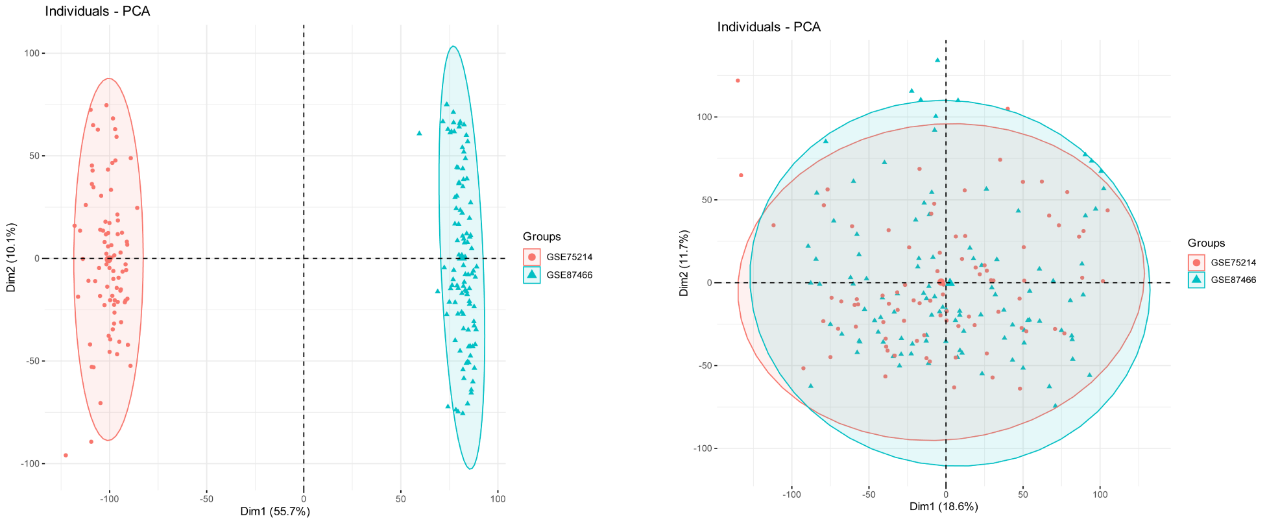


Figure S24 Batch correction using ComBat. Principal component analysis (PCA) was performed before and after batch correction.


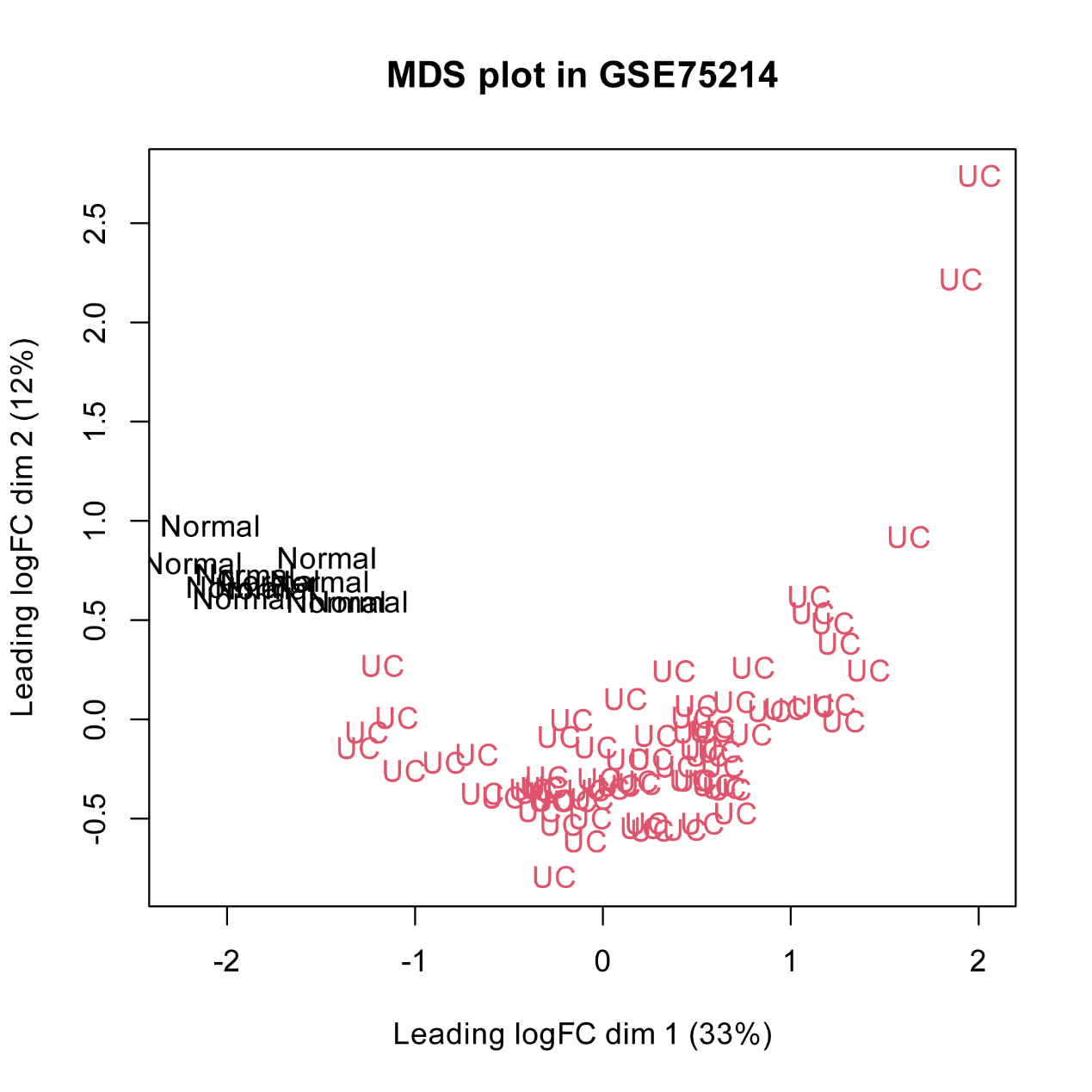


Figure S25 MDS plot of GSE75214.


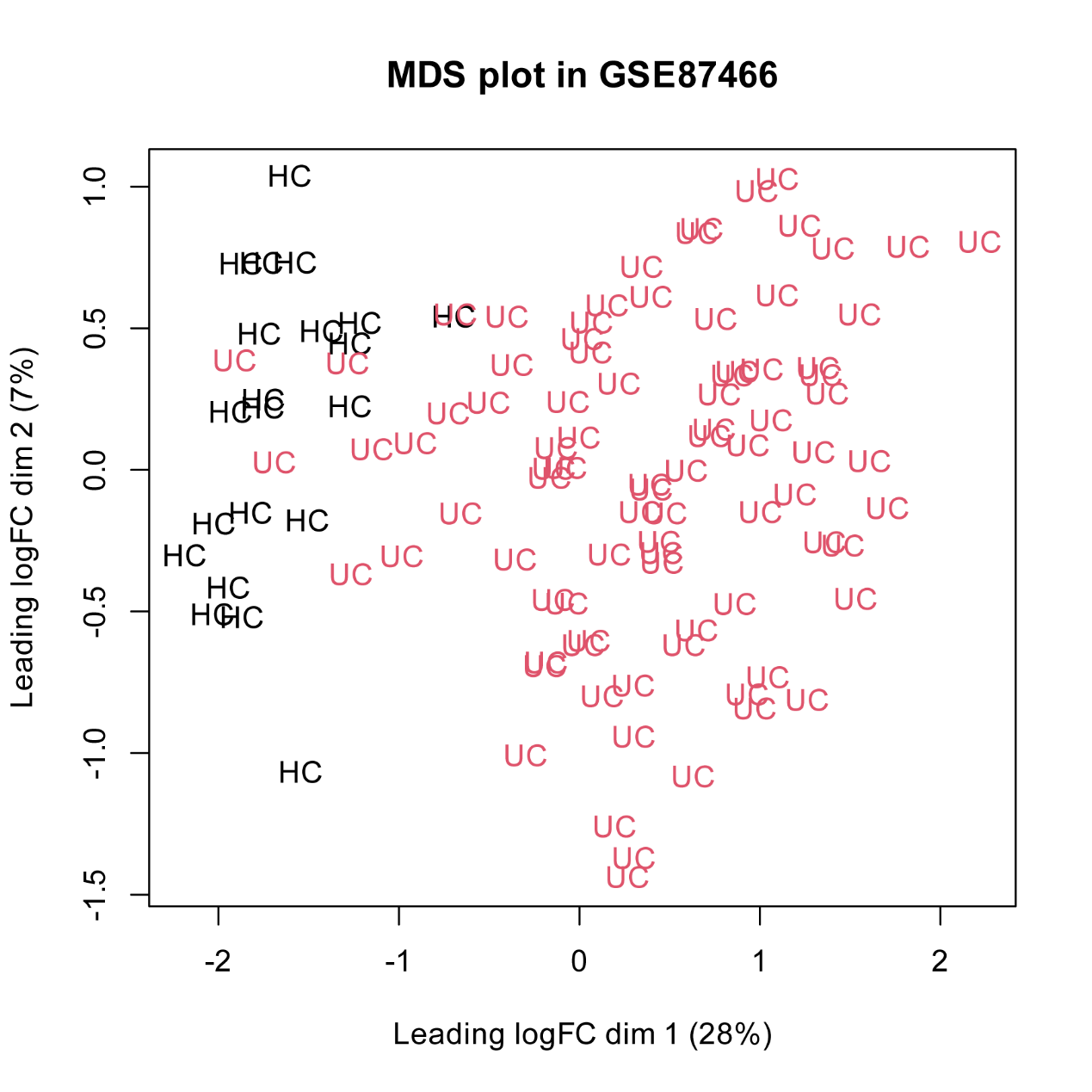


Figure S26 MDS plot of GSE87466.


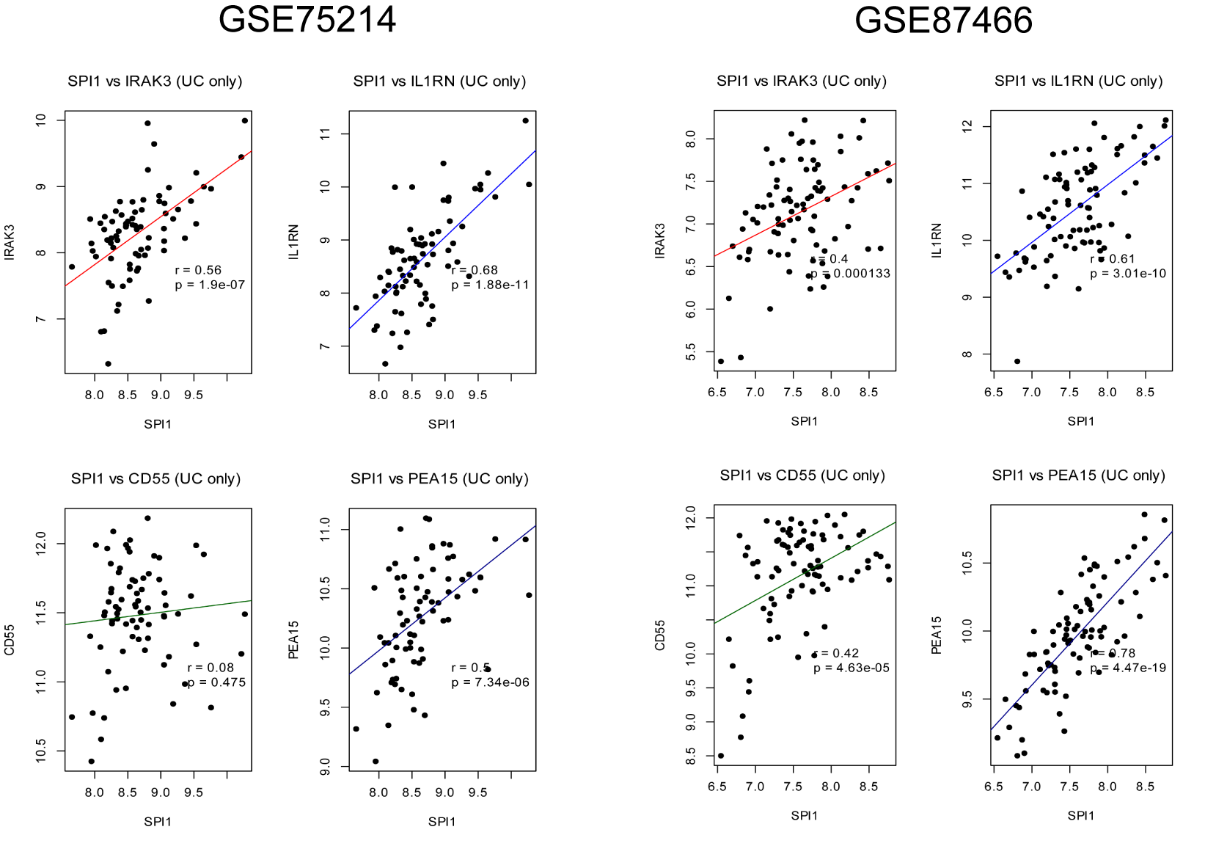


Figure S27 SPI1 expression showed a correlation with IRAK3, IL1RN, CD55 and PEA15 in human samples
